# Unifying network connectivity from geodesics to random walks via the random cluster model

**DOI:** 10.1038/s41467-026-72587-2

**Published:** 2026-06-27

**Authors:** Xiangyi Meng, Chenxuguang Zhu, Nicola Pedreschi, Erik Hormann, Duxin Chen, Gaogao Dong, Renaud Lambiotte

**Affiliations:** 1 Department of Physics, Applied Physics, and Astronomy, Rensselaer Polytechnic Institute, New York, Troy USA; 2 National Institute for Theory and Mathematics in Biology, Northwestern University and the University of Chicago, Illinois, Chicago USA; 3 Mathematical Institute, University of Oxford, Oxford, UK; 4 Department of Translational Medicine and Neuroscience, Università degli Studi di Bari, Bari, Italy; 5 School of Mathematics, University of Edinburgh, Edinburgh, UK; 6 Jiangsu Provincial Scientific Research Center of Applied Mathematics, Jiangsu, Nanjing China; 7 School of Mathematical Sciences, Jiangsu University, Jiangsu, Zhenjiang China

**Keywords:** Complex networks, Applied mathematics

## Abstract

Connectivity is a fundamental concept in network science, characterizing how interactions propagate through indirect pathways. While numerous connectivity metrics exist, such as shortest paths, effective resistance and minimum cut, each highlighting distinct structural features, their relationships remain largely fragmented. Here we show that these classical notions arise as limiting cases of a unified statistical-physics framework based on the random cluster (RC) model, which interprets connectivity as a principled synthesis of series and parallel transmission. By tuning its parameters, the RC model not only recovers classical connectivity measures but also extrapolates into unexplored regimes, leading to emergent notions of connectivity which yield practical tools for network learning tasks. In particular, RC connectivity naturally encodes the kinetics of growing paths, enhancing learning performance in dynamical settings such as epidemic spreading and neurodynamics. By linking structural, dynamical, and learning-based perspectives, RC connectivity establishes a general and interpretable foundation for the analysis of networked systems.

## Abstract (web summary)

Network connectivity is traditionally described by distinct measures like shortest paths or effective resistance. Here, authors introduce a unified statistical physics framework based on the random cluster model that encompasses these classical notions and defines a continuous family of new connectivity measures, offering a powerful tool to analyze structure and dynamics in complex networks.

## Introduction

Networks provide a powerful framework for studying interacting systems, from social dynamics to neural connectomes, and are crucial for understanding how influence spreads, information flows, and collective behaviors emerge^1,2^. At the heart of network science lies the concept of connectedness through walks and paths. This deceptively simple yet fundamental idea highlights the importance of indirect pathways in enabling communication between nodes not directly linked by an edge, allowing networks to be both sparse and connected. In an undirected setting, connectedness is a binary property: any pair of nodes is either connected or not, which partitions the network into connected components. To go beyond this binary view, a range of connectivity measures have been developed to quantify the strength of indirect connections and hence the proximity of nodes in the network. These include widely used similarity measures and kernels for downstream machine learning tasks such as link prediction and node classification^3^. Notable examples are: the shortest path distance, which considers only the minimal paths and overlooks longer alternatives; effective resistance, which reflects average commute times for random walkers and captures the influence of multiple parallel paths; and minimum cut connectivity, which quantifies the robustness of connectedness to link failures. Each measure highlights distinct aspects of connectivity, offering valuable but often discipline-specific insights and lacking a unified framework that reveals their underlying relationships.

**Fig. 1:**
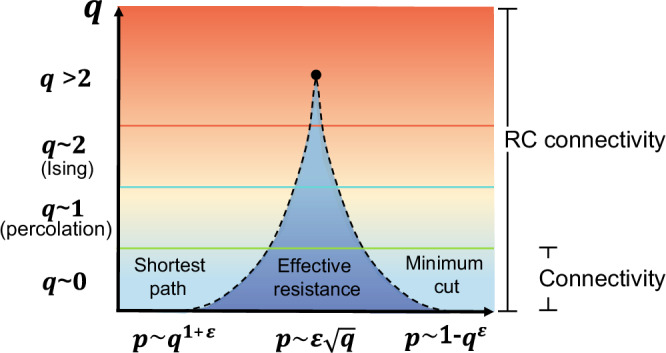
Unifying classical connectivity measures. This diagram illustrates how the random cluster (RC) model unifies discipline-specific connectivity measures into a single, continuous framework rooted in the fundamental concept of connected components, or simply connectedness. The model’s spectrum is governed by two parameters. The first, *p* ∈ [0, 1] (horizontal axis), sets the baseline probability for a link to be present. The second, *q* ∈ (0, *∞*) (vertical axis), is a statistical weight that favors configurations with more (*q* > 1) or fewer (*q* < 1) connected components compared to the baseline (*q* = 1). Note that the axes are not drawn to a quantitative scale. Classical network connectivity measures are recovered in the *q* → 0 limit, including three key regimes: (1) Effective resistance ($p \sim \epsilon \sqrt{q}$) defines connectivity through electrical flows and random walks. (2) Shortest path (*p* ~ *q*^1+*ϵ*^) focuses solely on optimal routes. (3) Minimum cut (*p* ~ 1 − *q*^*ϵ*^) measures robustness by identifying the network’s primary bottleneck. Here, *ϵ* is a positive number related to the typical link weight. For larger *q*, the RC model becomes equivalent to other cornerstone models, such as bond percolation (*q* = 1) and the Ising model of magnetism (*q* = 2). The RC model also exhibits a critical-like region ($p \sim \sqrt{q}$) that extends up to a threshold *q*_*c*_ ≳ 2 (qualitatively indicated by dashed lines), providing a powerful tool to explore complex network phenomena.

Here, we demonstrate that these three seemingly disparate connectivity measures can be brought together as specific limits of a broader statistical physics framework (Fig. 1): the random cluster (RC) model^4^. In all cases, connectivity is interpreted as the probability that two nodes belong to the same connected component in a percolation process. This unifying perspective offers both theoretical and practical benefits. Theoretically, it offers a novel path-based interpretation of established concepts, clarifying the fundamental series-versus-parallel logic that underpins different connectivity regimes. Practically, it enables the definition of a continuum of connectivity measures, which we term RC connectivity, by tuning the parameters of the RC model. This framework is particularly powerful for analyzing how network structure shapes system function^5–11^, offering a flexible, parameter-controlled family of methods to model the spread of influence between nodes. As an illustration, we show how RC connectivity outperforms traditional metrics in capturing correlations within epidemic and neurodynamical models, while also elucidating its relationship to community modularity and degree centrality. Together, these insights lay the foundation for a more unified theory of connectivity in networks, revealing how architecture governs behavior across diverse systems.

## Results

### Random cluster model for complex networks

Traditionally, the random cluster (RC) model is defined on regular lattice structures^12–14^. The RC model generalizes standard bond percolation^15–17^, a combinatorial and statistical physics model where each link of the lattice is independently present with a probability *p* (or absent with 1 − *p*)^12^. In bond percolation, a given configuration *ω* occurs with probability ${p}^{\left|\omega \right|}{\left(1-p\right)}^{\left|E\right|-\left|\omega \right|}$, where $\left|\omega \right|$ is the number of links in *ω* and $\left|E\right|$ is the total number of links in the original lattice. The RC model extends this occurrence probability through an extra parameter, *q* > 0, assigning an additional statistical weight to each *ω*:

1 $${p}^{\left|\omega \right|}{\left(1-p\right)}^{\left|E\right|-\left|\omega \right|}\to {p}^{\left|\omega \right|}{\left(1-p\right)}^{\left|E\right|-\left|\omega \right|}{q}^{c(\omega )}.$$

Here, *c*(*ω*) represents the number of connected components (clusters) within that specific configuration *ω*. This factor *q*^*c*(*ω*)^ explicitly couples the state of different links in a nontrivial way, effectively tuning the competition between clusters: for *q* > 1, configurations with more clusters are over-sampled; while for *q* < 1, configurations with fewer clusters are favored. Standard bond percolation is recovered by *q* = 1.

For our purpose, we extend the RC model to a positively weighted, undirected network ${{\mathcal{G}}}=(V,E)$ without self-loops. Each link *e* ∈ *E* is assigned a positive link weight *β*_*e*_ ∈ [0, *∞*) ∪ {*∞*}. To construct the RC model in terms of *β*_*e*_ in ${{\mathcal{G}}}$, we treat each weight *β*_*e*_ as a quantity of inverse temperature as in statistical spin models and convert it to a new, probability-like quantity *p*_*e*_ ∈ [0, 1] by invoking the Edwards–Sokal relation^18^:

2 $$1-{p}_{e}\equiv {e}^{-{\beta }_{e}}.$$

In this analogy, *p*_*e*_ can be treated as the heterogeneous probability that link *e* is present. The high-temperature limit (*β*_*e*_ → 0) corresponds to a low presence probability (*p*_*e*_ → 0), indicating vanishing coupling between the two end nodes of *e*.

With *p*_*e*_, we construct the RC model by writing down the partition function

3 $${Z}_{{{\rm{RC}}}}={\sum }_{\omega \in \Omega }P(\omega ){q}^{c(\omega )},$$

where $P(\omega )={\prod }_{e\in E}{p}_{e}^{{\omega }_{e}}{\left(1-{p}_{e}\right)}^{1-{\omega }_{e}}$ is the left-hand side of Eq. (1) after changing *p* → *p*_*e*_. Each configuration $\omega={({\omega }_{e})}_{e\in E}$ is a vector of binary variables where *ω*_*e*_ = 1 if link *e* is present and 0 otherwise, drawn from the space $\Omega={\{0,1\}}^{\left|E\right|}$.

Another key quantity for the RC model is the probability ${\mu }_{ij}^{(q)}$ that nodes *i* and *j* reside within the same cluster. This probability is given by the weighted sum over only those *ω*’s where *i* and *j* are connected:

4 $${\mu }_{ij}^{(q)}={Z}_{{{\rm{RC}}}}^{-1}{\sum }_{\omega \in \Omega | (i,j\, {{\rm{are}}}\,{{\rm{connected}}}\,{{\rm{in}}}\, \omega )}P(\omega ){q}^{c(\omega )},$$

which is normalized by ${Z}_{{{\rm{RC}}}}^{-1}$ and automatically satisfies the following properties for *q* ∈ (0, *∞*):

- Reflexivity: $${\mu }_{ii}^{(q)}=1$$.
- Transitivity: If ${\mu }_{ij}^{(q)},{\mu }_{jk}^{(q)} > 0$, then ${\mu }_{ik}^{(q)} > 0$.
- Symmetry: $${\mu }_{ij}^{(q)}={\mu }_{ji}^{(q)}$$.

Duality.—In the following, we consider a related quantity ${\nu }_{ij}^{(q)}$, which proves useful in its own right and is implicitly defined through

5 $${\mu }_{ij}^{(q)}=\frac{{\nu }_{ij}^{(q)}q}{{\nu }_{ij}^{(q)}q+\left(1-{\nu }_{ij}^{(q)}\right){q}^{2}}.$$

The latter can be interpreted as follows: consider that the original graph can be fully reduced to a two-node graph containing only *i* and *j*, with a single effective probability ${\nu }_{ij}^{(q)}$ defined for link {*i*, *j*}. There are only two configurations for this two-node graph: (1) with probability ${\nu }_{ij}^{(q)}$ the two nodes are connected, and *c*(*ω*) = 1; (2) with $1-{\nu }_{ij}^{(q)}$ the two nodes are disconnected, and *c*(*ω*) = 2. Then, Eq. (5) follows from the definition of ${\mu }_{ij}^{(q)}$ [Eq. (4)].

Both ${\mu }_{ij}^{(q)}\in [0,1]$ and ${\nu }_{ij}^{(q)}\in [0,1]$ are probability measures. They are also positively correlated for *q* ∈ (0, *∞*) and satisfy a duality relation, as can be seen from the transformation defined for *x* ∈ [0, 1] as

6 $${{\mathcal{M}}}(x)=\frac{1-x}{1+(q-1)x}.$$

This function ${{\mathcal{M}}}(x)$ is monotonically decreasing in *x*. It is also its own inverse, satisfying ${{\mathcal{M}}}({{\mathcal{M}}}(x))=x$. By Eq. (5), the transformation ${{\mathcal{M}}}$ provides a direct relationship between ${\mu }_{ij}^{(q)}$ and ${\nu }_{ij}^{(q)}$:

7 $$1-{\nu }_{ij}^{(q)}={{\mathcal{M}}}({\mu }_{ij}^{(q)}),\\ {\mu }_{ij}^{(q)}={{\mathcal{M}}}(1-{\nu }_{ij}^{(q)}).$$

We denote them as the *μ*- and *ν*-connectivity, respectively. Both are considered as the RC connectivity between any two nodes *i* and *j* in a network, providing a family of measures to capture the proximity of nodes depending on the values of *q* and *p*_*e*_.

### Classical connectivity measures

**Fig. 2:**
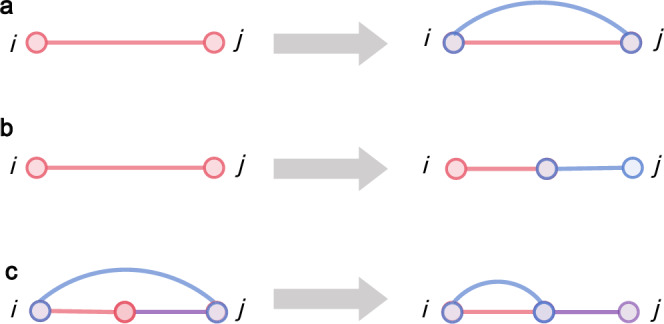
Key characteristics of connectivity. **a** Path multiplicity: Connectivity increases with the number of available paths, which provide alternative routes for transport between *i* and *j*. **b** Path efficiency: Connectivity decreases with longer paths, which impede flow much like how a signal attenuates over distance. **c** Path redundancy: Connectivity decreases with overlapping paths relative to disjoint paths, because shared links create vulnerabilities that can disrupt multiple routes simultaneously.

After introducing the RC connectivity, it is essential to examine how it compares to and extends traditional measures of network connectivity. A widely accepted view is that a meaningful connectivity measure should satisfy some—and should not contradict any—of the three fundamental principles outlined in Fig. 2^19^. In the following, we provide a brief overview of three classical connectivity metrics, each of which is widely used across disciplines and emphasizes different aspects of these guiding principles. Although originally developed in distinct fields and often applied in isolation, all three metrics, as we will show, emerge as limiting cases of the RC connectivity in the regime *q* → 0.

**Fig. 3:**
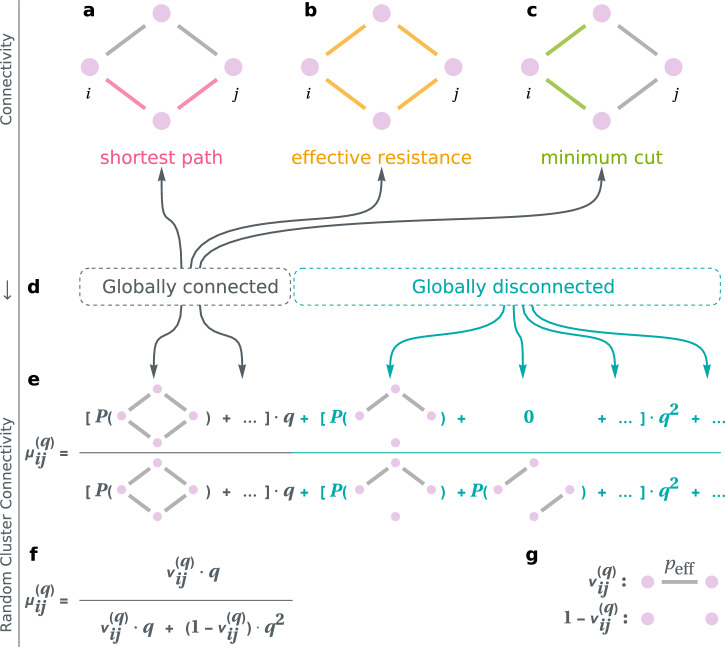
Connectivity from connectedness. **a**–**c** Classical connectivity measures capture contrasting network properties, from the most efficient geodesic path (pink) to “bottleneck” links between *i* and *j* (green), or their combination (orange). **d** The random cluster (RC) framework unifies these by interpreting each as the probability that nodes *i* and *j* belong to the same connected component. This unification is achieved by considering the RC ensemble of all possible configurations. In the *q* → 0 limit, the ensemble is dominated by globally connected configurations (black), from which classical measures emerge. **e** When *q* > 0, globally disconnected configurations (cyan) also contribute; their inclusion in the weighted average [in both the numerator and denominator of (**e**)] yields a new, continuous notion of connectivity: *μ*-connectivity ${\mu }_{ij}^{(q)}$. **f**–**g** The notion further leads to a dual measure, the *ν*-connectivity ${\nu }_{ij}^{(q)}$, which can be understood as (**g**) the link probability $p_{{\mbox{eff}}} \equiv \nu_{ij}^{(q)}$ defined for a single, effective link between nodes *i* and *j* in an equivalent two-node graph obtained by reducing the original using, e.g., series and parallel rules (Table 1).

**Effective resistance connectivity:** One powerful approach to quantifying the connectivity between two nodes *i* and *j* in a network is through the concept of effective resistance, denoted *R*_*ij*_. The network is interpreted as an electrical circuit in which each link *e* acts as a resistor assigned with a non-negative value: the resistance *r*_*e*_ ∈ [0, *∞*) (the absence of a link is treated as *r*_*e*_ → *∞*). Then, the effective resistance *R*_*ij*_ corresponds to the voltage difference between *i* and *j* when a unit current is injected at node *i* and extracted at node *j*. Mathematically, the effective resistance can be computed from the Moore-Penrose pseudoinverse of the graph Laplacian. The effective resistance has several elegant mathematical properties. Notably, it defines a metric on the set of nodes—i.e., it is symmetric, non-negative, satisfies the triangle inequality, and vanishes when *i* = *j*^20–22^. Moreover, the relative resistance between *i* and *j*, provided that the link {*i*, *j*} exists, is equal to the probability that this link is contained in a random spanning tree of the network. One of its most celebrated physical properties is captured by Rayleigh’s monotonicity principle^23,24^, which states that adding new links or reducing the resistance of existing ones (i.e., increasing link weights) can only decrease the effective resistance between any pair of nodes, and conversely, removing links or increasing their resistance increases *R*_*ij*_.

Intuitively, a small effective resistance between nodes *i* and *j* indicates that they are well connected—there exist many short or high-conductance paths between them—while a large effective resistance reflects sparse or long (low-weight) connectivity. In this way, *R*_*ij*_ captures a more integrated notion of distance than the shortest path, as it accounts for all possible paths and how they interrelate. The reciprocal, 1/*R*_*ij*_, known as effective conductance, serves as a natural measure of connectivity. Higher effective conductance implies stronger overall connectivity between two nodes and satisfies desirable connectivity principles as shown in Fig. 2^19^.

Beyond its electrical analogy, the effective resistance is also intimately linked to random walks on graphs: it is proportional to the expected commute time between *i* and *j*, i.e., the expected time for a random walker to travel from *i* to *j* and return^25^. This random walk interpretation underscores the utility of effective resistance in systems where flows do not follow the shortest paths. For example, it has found applications in ecology to model animal movement and gene flow across complex landscapes^26,27^. Furthermore, the one-way hitting time of random walks may also serve as a centrality measure within heterogeneous networks^28^.

**Shortest path connectivity:** A widely used and intuitive measure of connectivity is the shortest-path connectivity, defined as 1/*d*_*ij*_, the reciprocal of the geodesic distance (i.e., shortest path length) *d*_*ij*_ between nodes *i* and *j*. Here, the path length refers either to the number of links on the path in an unweighted network, or to the sum of their lengths *l*_*e*_ ∈ [0, *∞*)—a non-negative value defined for every link *e* in a weighted network. This measure clearly satisfies the path efficiency principle (outlined in Fig. 2)^19^, as a shorter path directly leads to a higher value of 1/*d*_*ij*_. In contrast, its relation to the path multiplicity and path redundancy principles is weaker, as it does not contradict them^29,30^. At best, it verifies them indirectly, as the presence of multiple paths increases the likelihood that at least one of them is short, even more so if the paths are independent. In many empirical networks, it is also common to find multiple shortest paths of the same length between two nodes, a phenomenon that has important implications for e.g., network efficiency and route decision-making^29,30^. Still, the shortest-path distance by definition considers only the minimal path and disregards longer alternatives^19,31^—even if they are numerous or important for real-world flow, which may limit its expressiveness in more complex dynamics.

The shortest-path distance is also a true metric, satisfying symmetry, non-negativity, and the triangle inequality. It is perhaps the most intuitive and commonly used dissimilarity measure in network analysis: the smaller the shortest-path distance between two nodes, the more similar or proximate they are considered to be. This makes it particularly relevant in domains where information or traffic follows (near-)optimal routes. A prime example is in computer networks, where routing protocols aim to minimize transmission costs or delays^32,33^.

**Minimum cut connectivity:** In graph theory, the edge-connectivity *λ*_*ij*_ between two nodes *i* and *j* is defined as the minimum total weight of links whose removal disconnects the two nodes^34^. This set of links is called a minimum cut. The minimum cut provides a natural measure of how robustly connected two nodes are: the more links that must be removed to sever their connection, the more strongly they are linked. Minimum-cut connectivity can also be interpreted in terms of independent paths, defined as paths that share no links. From this perspective, *λ*_*ij*_ quantifies the number of disjoint routes through which data or influence might propagate between two nodes^35^. A pair of nodes connected by many independent paths is more robustly connected than one joined by a single fragile route. By the celebrated max-flow/min-cut theorem, the value *λ*_*ij*_ also equals the maximum flow that can be routed from *i* to *j* when each link *e* is assigned a non-negative flow capacity: *c*_*e*_ ∈ [0, *∞*). Thus, the minimum-cut connectivity captures both structural and dynamic aspects of network connection, finding connections to algebraic connectivity through the well-known Cheeger inequalities^36^. Edge-connectivity clearly aligns with two key characteristics in Fig. 2, as multiple independent paths between nodes support higher potential flow (path multiplicity), while redundancy among paths weakens resilience to edge failures (path redundancy). In contrast, the dependency on path length (path efficiency) is only indirect, as a longer path between two nodes tends to increase the likelihood of encountering a low-capacity bottleneck link.

The interpretation of minimum-cut in terms of bottlenecks has natural relevance in applied domains such as management science, logistics, and operations research. Minimum-cut connectivity also reflects network robustness^37^. It reveals how vulnerable a connection is to disruption: the higher the value of *λ*_*ij*_, the more failure-tolerant the connection between *i* and *j*. In transportation networks, for example, *λ*_*ij*_ characterizes the number of traffic routes; the failure of all such routes would be necessary to halt the traffic.

### One connectivity to rule them all

Several previous works have explored the connections between connectivity measures. Much of the effort has focused on relating effective resistance to the shortest-path distance. On the one hand, while effective resistance captures all possible paths between two nodes, it can sometimes get lost in space, converging to meaningless values in large or sparse graphs^38,39^. On the other hand, the shortest-path distance is computationally simple and intuitive but ignores the multiplicity of shortest paths and longer, potentially critical alternative paths^19^. To reconcile these limitations, various families of interpolating distances have been proposed, designed to combine the robustness of effective resistance with the interpretability of shortest-path distance^40^. These interpolations are often controlled by a continuous parameter that adjusts the trade-off between favoring path length versus multiplicity^41^.

Another notable example is the relation between effective resistance and cut edges, expressed through bounds derived from the Nash–Williams criterion^42^. This connection allows one to estimate path multiplicity by identifying the bottleneck links. Furthermore, it has been shown that graph cuts and random walks can emerge as special cases of a broader, continuously parameterized optimization framework^43,44^.

Finally, effective resistance has been known to correspond to the ensemble of uniform spanning trees (USTs), which can be derived from the RC model^4,45^. This connection, which partly motivates our results below, has historically been limited to homogeneous networks where all link resistances are identical.

A central contribution of our work is to show that the three classical connectivity measures, effective resistance, shortest-path distance, and minimum cut, are all special cases of a more general framework—the RC connectivity—defined upon a heterogeneous graph Laplacian (Methods, “Derivation of RC connectivity from graph Laplacian”). Our main results are encapsulated in the following three theorems (proofs in Methods, “RC connectivity in the limit *q* → 0”):

#### Theorem 1

(Effective resistance) Let the probability associated with each link *e* in the network be ${p}_{e}=\sqrt{q}/{r}_{e}$, where *r*_*e*_ is the resistance of link *e*. Then, the inverse effective resistance 1/*R*_*ij*_ between any two nodes *i* and *j* is given by:

8 $${\lim}_{q\to 0}\frac{{\nu }_{ij}^{(q)}}{\sqrt{q}}=1/{R}_{ij}.$$

#### Theorem 2

(Shortest path) Let the probability associated with each link *e* in the network be ${p}_{e}={q}^{{l}_{e}+1}$, where *l*_*e*_ is the length of link *e*. Then, the inverse geodesic distance 1/*d*_*ij*_ between any two nodes *i* and *j* is given by:

9 $${\lim}_{q\to 0}\frac{{{\rm{ln}}}\, {q}}{{{\rm{ln}}}\, {\mu }_{ij}^{(q)}}=1/{d}_{ij}.$$

#### Theorem 3

(Minimum cut) Let the probability associated with each link *e* in the network be ${p}_{e}=1-{q}^{{c}_{e}}$, where *c*_*e*_ is the capacity of link *e*. Then, the maximum flow *λ*_*ij*_ between any two nodes *i* and *j* is given by:

10 $${\lim}_{q\to 0}\frac{{{\rm{ln}}}\left(1-{\nu }_{ij}^{(q)}\right)}{{{\rm{ln}}}\, q}={\uplambda }_{ij}.$$

These results reveal two fundamental insights. First, they offer a unified reinterpretation of classical connectivity measures in terms of a deeper concept: the connectedness of clusters (Fig. 3). Specifically, each measure arises as a distinct limit of the probability that two nodes belong to the same connected component in an associated RC model. Second, the framework extrapolates from existing notions into the unexplored regime where *q* > 0, defining a new and continuous family of connectivity measures. This family extends beyond specific instances of percolation (*q* = 1) and the Ising model (*q* = 2)—which themselves have rarely been applied as general network connectivity metrics. Crucially, the parameter *q* can be tuned to capture the structure or dynamics of empirical data, providing a principled and flexible tool for network analysis.

### Series and parallel rules

Here, we show that connectivity characteristics, from path efficiency to multiplicity (Fig. 2), can be made explicit using the so-called series and parallel reduction rules, a tool widely applied from circuit theory to quantum networks^46–49^. It is perhaps surprising that the RC connectivity is fully compatible with this series-parallel logic^4^: the rules strictly preserve ${\mu }_{ij}^{(q)}$ (or ${\nu }_{ij}^{(q)}$) between any nodes *i*, *j* remaining after reduction (Methods, “Series and parallel rules for the RC model”). Formally, for each link *e*, we may define two variables *u*_*e*_, *v*_*e*_ ∈ [0, 1] as functions of the probability measure *p*_*e*_, given by

11 $${u}_{e}={{\mathcal{M}}}(1-{v}_{e})={p}_{e}/\left[{p}_{e}+q\left(1-{p}_{e}\right)\right],\\ {v}_{e}=1-{{\mathcal{M}}}({u}_{e})={p}_{e},$$

which follow the exact same duality relation as the *μ*- and *ν*-connectivity in Eq. (7). We find that the series and parallel reduction rules can then be expressed concisely using *u*_*e*_ and *v*_*e*_, as shown in Table 1. Interestingly, both the series and parallel rules closely resemble those for standard bond percolation^47^, yet based on not one but two dual measures. When *q* = 1, we have *u*_*e*_ = *v*_*e*_ = *p*_*e*_, and Table 1 reduces to the percolation rules.

**Table 1:** Series and parallel rules for random cluster connectivity

	Random cluster model [*u*, *v* defined in Eq. (11)]
Series	$u{\prime}={{\rm{seri}}}\{{u}_{e},{u}_{f}\}={u}_{e}{u}_{f}$
Parallel	$v{\prime}={{\rm{para}}}\{{v}_{e},{v}_{f}\}=1-\left(1-{v}_{e}\right)\left(1-{v}_{f}\right)$

The series and parallel rules (Table 1) can be used to formalize the key connectivity characteristics in Fig. 2: We observe that

12 $${{\rm{seri}}}\{{u}_{e},{u}_{f}\} \le \min \{{u}_{e},{u}_{f}\},\\ {{\rm{para}}}\{{v}_{e},{v}_{f}\} \ge \max \{{v}_{e},{v}_{f}\},$$

which reflect the path efficiency and path multiplicity (Fig. 2). Further, combining the series and parallel rules [and employing Eq. (7) to switch between *u*_*e*_ and *v*_*e*_], we prove the following inequality:

13 $${{\rm{para}}}\{1-{{\mathcal{M}}}({{\rm{seri}}}\{{u}_{e},{u}_{f}\}),{v}_{g}\}\\ \ge 1-{{\mathcal{M}}}({{\rm{seri}}}\{{u}_{e},{{\mathcal{M}}}(1-{{\rm{para}}}\{{v}_{f},{v}_{g}\})\}),$$

which indicates that first-series (*e*, *f*) then-parallel (with *g*) always yields higher overall connectivity than first-parallel (*f*, *g*) then-series (with *e*). This behavior reflects the path redundancy (Fig. 2).

In the *q* → 0 limit, these rules reproduce intuitive rules for the classical connectivity metrics. Indeed, let ${p}_{e}=\sqrt{q}/{r}_{e}$, we recover the rules for effective resistance:

14 $${{\rm{seri}}}\{{r}_{e},{r}_{f}\} \simeq {r}_{e}+{r}_{f}+\ldots,\\ {{\rm{para}}}\{{r}_{e},{r}_{f}\} \simeq {\left(1/{r}_{e}+1/{r}_{f}\right)}^{-1}+\ldots ;$$

with ${p}_{e}={q}^{{l}_{e}+1}$, the geodesic distance:

15 $${{\rm{seri}}}\{{l}_{e},{l}_{f}\} \simeq {l}_{e}+{l}_{f}+\ldots,\\ {{\rm{para}}}\{{l}_{e},{l}_{f}\} \simeq \min \{{l}_{e},{l}_{f}\}+\ldots ;$$

and with ${p}_{e}=1-{q}^{{c}_{e}}$, the maximum flow:

16 $${{\rm{seri}}}\{{c}_{e},{c}_{f}\} \simeq \min \{{c}_{e},{c}_{f}\}+\ldots,\\ {{\rm{para}}}\{{c}_{e},{c}_{f}\} \simeq {c}_{e}+{c}_{f}+\ldots .$$

### Implementing RC connectivity

Having defined the RC connectivity ${\mu }_{ij}^{(q)}$ (and ${\nu }_{ij}^{(q)}$) in Eqs. (4) and (5), we now outline the implementation procedure of the RC connectivity for real networks:

1. Construct the weighted, undirected graph Laplacian of the network, where each link *e* carries a weight *β*_*e*_. (For unweighted networks, we may simply set *β*_*e*_ = 1 for existing links and zero otherwise.)
2. Select the parameter *q*.
3. Calculate the *μ*-connectivity ${\mu }_{ij}^{(q)}$ for every node pair (*i*, *j*)—either (a) via Monte-Carlo simulation of Eq. (4) after converting *β*_*e*_ to a probability measure *p*_*e*_ through $1-{p}_{e}\equiv {e}^{-{\beta }_{e}}$ [Eq. (2)], or (b) directly from the graph Laplacian (Methods, “Derivation of RC connectivity from graph Laplacian”).

While there is no rigid rule for what *q* value should be selected, the parameter *q* essentially acts as a proxy for path interactions during path growth. Therefore, turning on *q* > 0 is appropriate when we expect the connectivity is governed by strong interactions between parallel paths (Fig. 2). Notably, for sufficiently large *q* (typically *q* ≳ 2), the model becomes less insightful as the critical region vanishes due to the onset of discontinuous transitions (Fig. 1). Consequently, the regime 0 < *q *≤ 2 offers a reasonable choice for analyzing real complex networks.

From a practical standpoint, given an ensemble of Monte-Carlo realizations, it requires $O({\left|V\right|}^{2})$ time per configuration *ω* to calculate the connectedness between every pair of nodes *i* and *j* and to determine the number of connected components *c*(*ω*). Depending on available memory and CPU resources, multiple realizations can be executed simultaneously through parallel processing. After this step, it is computationally inexpensive to sweep through different *q* values. One can simply adjust the weights *q*^*c*(*ω*)^ in Eqs. (3) and (4) without regenerating the ensemble. Therefore, for practical applications, it can be beneficial to sweep and identify the optimal *q* value rather than relying on an arbitrary guess.

Next, we demonstrate that implementing RC connectivity with *q* > 0 circumvents the limitations of traditional measures in the *q* → 0 limit, especially for analyzing network dynamical models.

### RC connectivity captures general network dynamics

**Fig. 4:**
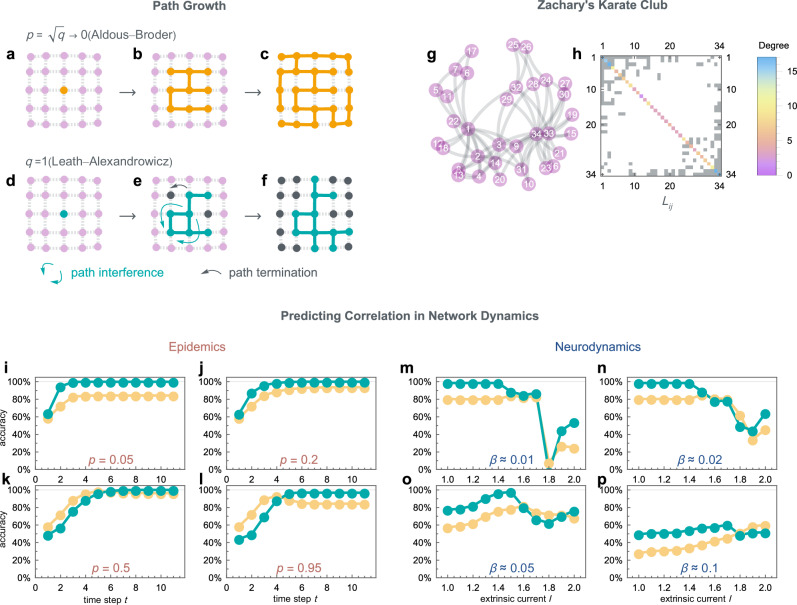
Path growth captured by RC connectivity. **a**–**f** RC configurations can be interpreted as outcomes of path growth depending on the parameter *q*. **a**–**c** In the limit ${p}_{e}\propto \sqrt{q}\to 0$, the dominant configurations are uniform spanning trees, featuring a unique path between every two nodes in the network. These configurations can be generated by the Aldous--Broder algorithm. **d**–**f** Conversely, for a general *q*, a unique path between two nodes is not guaranteed. Instead, paths can multiply and interact---creating redundancy---or they can terminate prematurely before spanning the entire network during their growth. Specifically, for *q* = 1 these paths can be generated by the Leath--Alexandrowicz algorithm. The relevance of path interference and termination is further explored for simulating dynamical models on a network and predicting correlation along paths using the RC connectivity. **g** Zachary’s karate club, a commonly studied network structure, with its (**h**) graph Laplacian *L*_*ij*_. **i**–**l** Assessment of how well different connectivity measures capture correlations between nodes in a Susceptible-Infected-Recovered epidemic model. Higher accuracy indicates better prediction of the correlations, shown for varying disease transmission probabilities: (**i**)*p*_*e*_ = 0.05, (**j**)*p*_*e*_ = 0.2, (**k**)*p*_*e*_ = 0.5, and (**l**)*p*_*e*_ = 0.95. **m**–**p** Same assessment for a FitzHugh--Nagumo neurodynamics model, with varying signal diffusion rates: (**m**)*β*_*e*_ ≈ 0.01, (**n**)*β*_*e*_ ≈ 0.02, (**o**)*β*_*e*_ ≈ 0.04, and (**p**)*β*_*e*_ ≈ 0.06. Green dots represent RC connectivity with *q* = 1, and yellow dots represent the effective resistance. Each physical value of *p*_*e*_ (or *β*_*e*_) directly maps to the parameter *p* we used for calculating ${\mu }_{ij}^{(q=1)}$.

To begin with, building on the well-known equivalence between bond percolation and spreading processes^50^, we argue that the RC model allows a path-growing interpretation, where connections are built by dynamically growing paths. To understand this kinetic view, we consider the limiting case of the effective resistance connectivity [Eq. (8)], focusing on a homogeneous network with ${p}_{e}\propto \sqrt{q}\to 0$. In this regime, the dominant configurations are USTs (Methods, “Random-walk interpretation of effective resistance”). These USTs can be generated kinetically by the Aldous–Broder algorithm^51,52^, which randomly grows a single UST that spans the entire network, ensuring that there is one and only one path connecting any two nodes (Fig. 4a–c).

This uniqueness of paths can be further illustrated as follows: consider a random-cluster configuration *ω* where the state of all links except for one, *e*, has been determined. The probability that link *e* is present is then conditionally given by ref. ^4^

17 $${{\rm{presence}}}\,{{\rm{probability}}}=\left\{\begin{array}{ll}{u}_{e},& {{\rm{if}}}\,e\,{{\rm{is}}}\,{{\rm{a}}}\,{{\rm{cut}}}{\mbox{-}}{{\rm{edge}}}\,{{\rm{of}}}\,\omega \cup \{e\};\hfill \\ {v}_{e},& {{\rm{if}}}\,e\,{{\rm{is}}}\,{{\rm{not}}}\,{{\rm{a}}}\,{{\rm{cut}}}{\mbox{-}}{{\rm{edge}}}\,{{\rm{of}}}\,\omega \cup \{e\},\end{array}\right.$$

where *u*_*e*_ and *v*_*e*_ are given by Eq. (11). This formula essentially states that if link *e*’s endpoints are already connected in *ω*, such that adding *e* creates a cycle, then the probability of *e*’s presence is *v*_*e*_. Otherwise, if adding *e* bridges two previously disconnected components in *ω*, then its probability of presence is adjusted to *u*_*e*_. In the ${p}_{e}\propto \sqrt{q}\to 0$ limit, we recover *u*_*e*_ → 1 and *v*_*e*_ → 0 [Eq. (11)]. This implies that a necessary connection is always formed while a redundant one is rejected, which is the precise kinetic definition of a spanning tree.

As *q* (and ${p}_{e}\propto \sqrt{q}$) increases from zero, we expect that *u*_*e*_ decreases from one and *v*_*e*_ increases from zero, respectively. We immediately see two changes:

- First, since *u*_*e*_ < 1, a connecting path is not guaranteed to form; it may spontaneously terminate.
- Second, since *v*_*e*_ > 0, multiple paths to a node can exist. This multiplicity is not a simple superposition but reflects an interference between paths: they collectively trigger a binary response of connectedness, determining if the target node becomes connected. Once a node is connected, it absorbs these incoming influences and can propagate new paths to its neighbors, losing the historical information of the incoming paths. This is analogous to rumor propagation, where a person hearing a rumor from multiple sources becomes informed and passes it without retaining the history of how the information arrived.

Specifically, for *q* = 1, we recover *u*_*e*_ = *v*_*e*_ = *p*_*e*_, corresponding to standard bond percolation, which can be described by the Leath–Alexandrowicz algorithm^53,54^. In this process, a percolating cluster—which does not necessarily span the entire network—emerges from the iterative wetting of its advancing front, which is itself formed by interfering paths (Fig. 4d–f).

Thus, increasing *q* fundamentally leads to the possibility of path interactions. This makes the model inherently more realistic for many real-world dynamics. For instance, in epidemic spreading, multiple message-passing routes to an individual can interact rather than superposing independently^55^. Similarly, in neural networks, action potentials converging on a neuron can collide or annihilate, leading to complex responses rather than simple pass-through^56^. To test the ability of RC connectivity to capture such effects, we selected two network dynamical models: (1) the Susceptible-Infected-Recovered (SIR) epidemic model, and (2) the FitzHugh–Nagumo (FHN) neuronal model. Through simulations of these models (Methods, “Correlations in network dynamics”), our goal is to assess how well RC connectivity captures the emergent correlations between nodes.

Results.—We define two measures of dynamic correlation: Ξ_*ij*_(*t*) for the SIR model and ${{{\mathcal{V}}}}_{ij}(I)$ for the FHN model. Specifically, Ξ_*ij*_(*t*) represents the probability that node *i* is in the recovered state at time *t*, given that node *j* was the initial seed node; and ${{{\mathcal{V}}}}_{ij}(I)$ is defined as the peak-to-peak value of the voltage oscillations of node *i*, given that node *j* was the site of the sustained external current *I*. To quantify how accurately the RC connectivity measure ${\mu }_{ij}^{(q)}$ predicts Ξ_*ij*_(*t*) and ${{{\mathcal{V}}}}_{ij}(I)$, we calculated the Spearman’s rho correlation coefficient between ${\mu }_{ij}^{(q)}$ and Ξ_*ij*_(*t*), and also between ${\mu }_{ij}^{(q)}$ and ${{{\mathcal{V}}}}_{ij}(I)$. The results remain identical if replacing ${\mu }_{ij}^{(q)}$ by ${\nu }_{ij}^{(q)}$, as Spearman’s rho is rank-based (and thus used here to avoid assumptions of linearity). The network was chosen as Zachary’s karate club (Fig. 4g, h), the size of which allows ${\mu }_{ij}^{(q)}$ to be calculated analytically without Monte-Carlo simulation. We start by setting *q* = 1, so that the RC model is equivalent to standard bond percolation (see Methods for other *q-*values). We see that the resulting Spearman’s rho values are consistently positive and significant, indicating that ${\mu }_{ij}^{(q=1)}$ effectively captures the strength of correlations in the network. For comparison, we also evaluated 1/*R*_*ij*_ [Eq. (8)] by computing its Spearman’s rho with the correlation functions Ξ_*ij*_(*t*) and ${{{\mathcal{V}}}}_{ij}(I)$. In general, ${\mu }_{ij}^{(q=1)}$ provides more accurate prediction compared to 1/*R*_*ij*_.

Specifically, for the SIR model (Fig. 4i–l), ${\mu }_{ij}^{(q=1)}$ shows excellent predictive power for sufficiently long times (*t*≥6). In this regime, the Spearman’s rho approaches unity, reflecting the known convergence of long-time SIR dynamics to percolation statistics^50^. The primary scenario where 1/*R*_*ij*_ shows a higher accuracy is when the infection probability *p* is high, but the observation time *t* is short. This is attributable to early-time SIR dynamics often being well-approximated by a diffusion-like process^57^, which is more closely related to random walks and thus to the effective resistance measure.

For the FHN model (Fig. 4m–p), ${\mu }_{ij}^{(q=1)}$ generally exhibits superior performance to 1/*R*_*ij*_ when the external current *I* is in the range of 1.0 to 1.5. However, for *I* > 1.5, the Spearman’s rho values for both ${\mu }_{ij}^{(q=1)}$ and 1/*R*_*ij*_ decrease. Examination of the temporal dynamics in this regime suggests this decline is due to localized network activity, where only a small fraction of nodes become effectively stimulated into oscillation, thereby weakening the correlation with global structural measures.

Taken together, we show that incorporating the RC connectivity (specifically, *q* > 0) can be useful for capturing correlations in network dynamical models. This underscores the potential for broader applications of our results.

### RC connectivity is dominated by degree and modularity

Given the definition of the RC connectivity, a natural question that arises is what network properties may cause higher values of RC connectivity between certain pairs of nodes. Here, we investigate the competing effects of two possible mechanisms:

1. On the one hand, in modular networks, pairs of nodes within the same community are expected to exhibit, on average, higher RC connectivity compared to pairs located in different communities, given the higher number of paths between them.
2. On the other hand, in heterogeneous networks, high-degree nodes are expected to possess greater RC connectivity. This is since as individual links are randomly removed, nodes with a higher degree retain a statistical advantage in maintaining connectivity to other nodes.

**Fig. 5:**
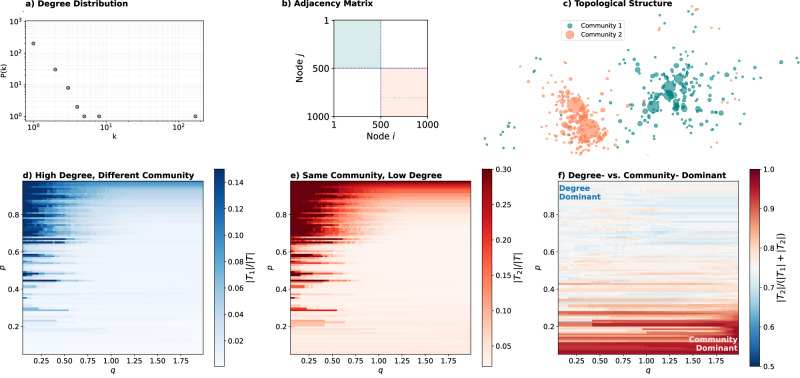
Dominant factors of RC Connectivity. **a** Power-law degree distribution of the network. **b** Adjacency matrix with 1000 nodes grouped into two communities. **c** Each community is composed of a few hubs and a majority of low-degree nodes. **d**–**f** Denote *T* as the set of the top 1% of node pairs ranked by *μ*_*ij*_. Within *T* two subsets are identified: *T*_1_ (high-degree inter-community pairs) and *T*_2_ (low-degree intra-community pairs). The fractional sizes (**d**) ∣*T*_1_∣/∣*T*∣ and (**e**) ∣*T*_2_∣/∣*T*∣, as well as (**f**) their relative dominance ∣*T*_2_∣/(∣*T*_1_∣ + ∣*T*_2_∣), are shown.

In other words, pairs of nodes with high degrees or those belonging to the same community are expected to exhibit higher RC connectivity. To verify this hypothesis, we considered an ensemble of synthetic networks generated by a degree-corrected stochastic block model^58^, which are both modular (two communities) and scale-free (with power-law exponent *γ* ≈ 1.3), as shown in Fig. 5a–c. We also assigned a uniform link weight *β*_*e*_ ∈ [0, *∞*) for each link.

Results.—For each network realization, we computed the RC *μ*-connectivity *μ*_*ij*_ for all distinct pairs of nodes *i* ≠ *j* with *q* = 1 for an unweighted network *β*_*e*_ = 1 (corresponding to *p*_*e*_ = 1 − *e*^−1^ ≈ 0.632). We then identified the set *T* consisting of the pairs $(i^{\prime},j^{\prime} )$ whose connectivity ranks in the top 1% of the entire distribution. Averaging over the ensemble, we find that as much as 63.0% of these high-connectivity pairs $(i^{\prime},j^{\prime} )\in T$ belong to the same community, denoted by $m(i^{\prime} )=m(j^{\prime} )$, and as much as 39.8% have higher-than-average degrees, satisfying ${d}_{i^{\prime} }+{d}_{j^{\prime} }\ge 2\langle d\rangle$ where 〈*d*〉 is the average node degree of the network. These findings suggest that both community structure and the underlying degree distribution are the primary determining factors of high RC connectivity.

In addition, we extended our analysis by adding link weight *β*_*e*_ to distinguish the regimes where high degrees dominate RC connectivity from those where modularity prevails. To this end, we consider the following two subsets of *T*:

18 $$\begin{array}{rcl}{T}_{1} &=& \{(i^{\prime},j^{\prime} )\in T\,\,| \,\,{d}_{i^{\prime} }+{d}_{j^{\prime} }\ge 2\langle d\rangle,\,{{\rm{but}}}\,m(i^{\prime} )\,\ne \,m(j^{\prime} )\};\\ {T}_{2} &=& \{(i^{\prime},j^{\prime} )\in T\,\,| \,\,m(i^{\prime} )=m(j^{\prime} ),\,{{\rm{but}}}\, {d}_{i^{\prime} }+{d}_{j^{\prime} } < 2\langle d\rangle \}.\end{array}$$

In other words, *T*_1_ is the subset of high-connectivity pairs that have higher-than-average degrees but reside in different communities (Fig. 5d), while *T*_2_ is the subset of high-connectivity pairs that share the same community but have lower-than-average degrees (Fig. 5e). We observe that in the limits of small *q* (*q* → 0) or strong link weights (*p*_*e*_ → 1), the ratios ∣*T*_1_∣/∣*T*∣ and ∣*T*_2_∣/∣*T*∣ increase. This suggests that the contributions of the two characteristics (high degree vs. same community) become increasingly distinct. Consequently, high-connectivity pairs in these regimes tend to be dominated by a single characteristic rather than a combination of both.

Further, we calculate the ratio ∣*T*_2_∣/(∣*T*_1_∣ + ∣*T*_2_∣) (Fig. 5f), finding that in the low-weight regime (small *p*_*e*_), the ratio ∣*T*_2_∣/(∣*T*_1_∣ + ∣*T*_2_∣) is close to 1, indicating that intra-community links are the primary drivers for high-connectivity pairs. This is because, as *p*_*e*_ → 0, the network is highly sparsified; since internal community links are more numerous, the few connecting paths are overwhelmingly contained within community boundaries. Consequently, pairs in *T*_2_ maintain connectivity while pairs in *T*_1_ (spanning different communities) lose their connectedness entirely. In the high-weight regime (large *p*_*e*_), we observe a transition where the ratio decreases, and the network becomes closer to degree dominant. This is because, as *p*_*e*_ → 1, the graph becomes denser; in this limit, global connectedness is high for all pairs, and the top-tier RC connectivity values are increasingly determined by the raw number of links connected to the nodes (degree). Thus, the influence of the global hubs (*T*_1_) begins to balance the local community structures (*T*_2_), pushing the transition boundary toward the blue regime.

## Discussion

Connectivity lies at the heart of modern network science, underpinning our understanding of how interactions unfold across complex systems. Yet, traditional measures often encode rigid, ad hoc notions of proximity, failing to capture the rich diversity of connectivity patterns observed in real-world networks. In this work, we have introduced a flexible framework, the RC connectivity, that unifies and generalizes classical metrics through the lens of the RC model^4^. This approach allows for a principled interpolation between competing connectivity principles based on path multiplicity, efficiency and redundancy, and offers tunable control over series versus parallel communication pathways between nodes.

Our formulation not only clarifies the theoretical relationships between classical connectivity measures—shortest paths, effective resistance, and min cuts—but also suggests concrete mechanisms for incorporating RC connectivity into modern graph learning architectures. In particular, RC connectivity defines a parametric, probabilistic geometry on graphs that generalizes the notion of a node’s neighborhood from fixed, hop-based sets to task-adaptive connectivity kernels. This perspective is related in spirit to approaches such as node2vec^59^, where biased random walks controlled by learnable parameters interpolate between breadth-first and depth-first exploration strategies to optimize downstream performance. While node2vec modulates how neighborhoods are sampled, RC connectivity instead parametrizes what it means for nodes to be connected, making connectivity itself a tunable and potentially learnable object. Within this framework, RC-derived statistics—such as pairwise connectivity probabilities, expected cluster sizes, or multiscale connectivity profiles—can be used both to guide message passing in graph neural networks^60^ and to construct positional encodings that capture global structure beyond, for example, Laplacian spectra. Embedding these physics-informed descriptors into learning architectures provides a path toward more expressive and interpretable representations, capable of capturing latent pathways, structural robustness, and dynamical modes of interaction across complex systems.

Ultimately, our key contribution is to define a generalizable and interpretable notion of connectivity that bridges structural and dynamic perspectives. This flexible view provides a foundation for learning richer, context-aware node and link representations, supporting the development of models that are not only more accurate but also more aligned with the mechanisms governing complex systems. As such, RC connectivity offers a new paradigm for understanding and predicting networked phenomena across disciplines.

## Methods

### Derivation of RC connectivity from graph Laplacian

The random cluster (RC) model has deep connections with statistical physics^45^. However, it has seen rare application in network science^57^. Networks, especially their dynamical properties, are typically characterized by the graph Laplacian

19 $${L}_{ij}={\sum }_{k}{\beta }_{ik}{\delta }_{ij}-{\beta }_{ij},$$

where *β*_*ij*_ ≡ *β*_*ji*_ is the weight of the link between nodes *i* and *j* (with *β*_*ij*_ = 0 if no link exists). The diagonal element *L*_*ii*_ = ∑_*k*_*β*_*ik*_ is the weighted degree of node *i*, while the non-diagonal elements are minus the link weights.

Our objective is to formulate the RC model directly in terms of this Laplacian, *L*_*ij*_. We present our main results first: the partition function *Z*_RC_ and the connectivity ${\mu }_{ij}^{(q)}$, defined by Eqs. (3) and (4) in the main paper, can be rewritten directly in terms of *L*_*ij*_ as

20 $${Z}_{{{\rm{RC}}}}={\sum }_{b\in {{\mathcal{B}}}}{\prod }_{i,j=1}^{\left|V\right|}{\left({e}^{-{L}_{ij}/2}-1\right)}^{{b}_{ij}}{q}^{c(b)}$$

and

21 $${\mu }_{ij}^{(q)}=1-\frac{q}{1-q}\left(\frac{\partial }{\partial {L}_{ii}}+\frac{\partial }{\partial {L}_{jj}}-\frac{\partial }{\partial {L}_{ij}}-\frac{\partial }{\partial {L}_{ji}}\right){{\rm{ln}}}\,{Z}_{{{\rm{RC}}}}.$$

Here, ${{\mathcal{B}}}={\{0,1\}}^{\left|V\right|\times \left|V\right|}$ denotes the complete set of $\left|V\right|\times \left|V\right|$ Boolean matrices, and *c*(*b*) denotes the number of weakly connected components of the directed graph represented by the configuration *b*, which is understood as an unweighted, bare adjacency matrix. This expression is instrumental in proving the relationship between ${\mu }_{ij}^{(q)}$ and other traditional network connectivity measures.

#### Derivation

To derive *Z*_RC_ and ${\mu }_{ij}^{(q)}$ from the graph Laplacian, we start by noting that the RC model represents an analytical continuation of the Potts model, a statistical physics model used to understand phase transitions and interactions in physical systems^45^. The partition function of the Potts model is given by

22 $${Z}_{{{\rm{Potts}}}}={\sum }_{\sigma \in \Sigma }\left[{\prod }_{e\in E}\exp \left({\beta }_{e}{\delta }_{e}(\sigma )\right)\right],$$

where each realization $\sigma=({\sigma }_{1},{\sigma }_{2},\cdots \,,{\sigma }_{\left|V\right|})$ is sampled from $\Sigma={\{1,2,\cdots \,,q\}}^{\left|V\right|}$. Given an undirected link *e* = {*i*, *j*}, the indicator function *δ*_*e*_(*σ*) is equal to one when *σ*_*i*_ = *σ*_*j*_ and otherwise zero when *σ*_*i*_ ≠ *σ*_*j*_. The inverse temperature *β*_*e*_ ∈ [0, *∞*) will serve as the positive link weight measure of the underlying graph.

The two models are constructed on different sample spaces: the RC model is sampled on links (*ω* ∈ *Ω*), while the Potts model is sampled on nodes (*σ* ∈ *Σ*). Nevertheless, through the Edwards–Sokal representation^18^, the two models are related by

23 $${Z}_{{{\rm{RC}}}}={e}^{-{\sum }_{e}\,{\beta }_{e}}{Z}_{{{\rm{Potts}}}},$$

under the change of variables ${p}_{e}\equiv 1-{e}^{-{\beta }_{e}}$.

Moreover, in the Potts model, the probability of having two nodes *i*, *j* in the same state is

24 $${p}_{{{\rm{Potts}}}}({\sigma }_{i}={\sigma }_{j})={Z}_{{{\rm{Potts}}}}^{-1}{\sum }_{\sigma \in \Sigma }{\delta }_{{\sigma }_{i}{\sigma }_{j}}\left[{\prod }_{e\in E}\exp \left({\beta }_{e}{\delta }_{e}(\sigma )\right)\right].$$

This probability is related to the probability of the same two nodes *i* and *j* being connected in the corresponding RC model^4^,

25 $${\mu }_{ij}^{(q)}=\frac{{p}_{{{\rm{Potts}}}}({\sigma }_{i}={\sigma }_{j})-1/q}{1-1/q}.$$

We have defined ${\mu }_{ij}^{(q)}$ as the RC connectivity (*μ*-connectivity) in the main paper. This established connection between the RC connectivity and the spin correlation *p*_Potts_(*σ*_*i*_ = *σ*_*j*_) in the Potts model allows us to write ${\mu }_{ij}^{(q)}$ as a derivative of *Z*_RC_ with respect to *β*_*e*_. To this end, we expand the product over undirected links *e* in *Z*_RC_ [Eq. (3) in the main paper] into a product over nodes $1\le i < j\le \left|V\right|$. This can be done by defining the weighted adjacency matrix *β*_*ij*_ = *β*_*ji*_ = *β*_*e*_ for every undirected link *e* = {*i*, *j*} ∈ *E* and *β*_*ij*_ = *β*_*ji*_ = 0 for every {*i*, *j*} ∉ *E*. The partition function will remain unchanged after summing over {*i*, *j*} ∉ *E*, as the exponential function $\exp {\beta }_{ij}$ will be equal to unity, which does not affect the partition function.

We also define a set of *q* vectors, **e**^1^, **e**^2^, ⋯  , **e**^*q*^, by

26 $${{{\bf{e}}}}^{\alpha }\cdot {{{\bf{e}}}}^{\beta }=\frac{{\delta }^{\alpha \beta }-1/q}{1-1/q},\,\alpha,\beta=1,2,\cdots \,,q.$$

Geometrically, these vectors correspond to the coordinates of the *q* vertices of a $\left(q-1\right)$-simplex. The inner product between two vectors can be understood as the physical coupling between two spins that point to the directions of these vectors. As a result, the partition function *Z*_RC_ can be rewritten as

27 $${Z}_{{{\rm{RC}}}}=	 {e}^{-{\sum }_{i < j}{\beta }_{ij}}{Z}_{{{\rm{Potts}}}} \\=	 \mathop{\sum }_{\{{{\bf{s}}}\}}\exp {\sum }_{i < j}{\beta }_{ij}\left(\frac{q-1}{q}{{{\bf{s}}}}_{i}\cdot {{{\bf{s}}}}_{j}+\frac{1}{q}-1\right) \\=	 {\sum }_{\{{{\bf{s}}}\}}\exp {\sum }_{i < j}\frac{q-1}{q}{\beta }_{ij}\left({{{\bf{s}}}}_{i}\cdot {{{\bf{s}}}}_{j}-1\right),$$

where the new summation over {**s**} enumerates all *q* possible states **s**_*i*_ ∈ {**e**^1^, **e**^2^, ⋯  , **e**^*q*^} at every node *i*. This allows us to write down the two-point function^4^,

28 $$\left\langle {{{\bf{s}}}}_{i}\cdot {{{\bf{s}}}}_{j}\right\rangle=\frac{1}{{Z}_{{{\rm{Potts}}}}}\frac{\partial {Z}_{{{\rm{Potts}}}}}{\partial {\beta }_{ij}}.$$

Note that $\left\langle {{{\bf{s}}}}_{i}\cdot {{{\bf{s}}}}_{j}\right\rangle$ is actually equal to ${\mu }_{ij}^{(q)}$ by comparing Eq. (26) with Eq. (25). Therefore, the RC connectivity is exactly the two-point function of the corresponding Potts model for integer *q *≥ 2. Further expanding Eq. (28) yields

29 $${\mu }_{ij}^{(q)}=1+\frac{q}{q-1}\frac{1}{{Z}_{{{\rm{RC}}}}}\frac{\partial {Z}_{{{\rm{RC}}}}}{\partial {\beta }_{ij}}.$$

Next, we rewrite Eq. (27) as

30 $${Z}_{{{\rm{RC}}}}={\sum }_{\{{{\bf{s}}}\}}\exp {\sum }_{i\ne j}\frac{q-1}{q}\frac{{\beta }_{ij}}{2}\left({{{\bf{s}}}}_{i}\cdot {{{\bf{s}}}}_{j}-1\right),$$

where the factor of 1/2 is because both *β*_*ij*_ and *β*_*ji*_ are now counted in the sum over *i* and *j*. The physical interpretation of having both *β*_*ij*_ and *β*_*ji*_ in the RC model is straightforward: it corresponds to having two identical, undirected links for every pair of adjacent nodes, where the first link’s presence probability is ${p}_{ij}\equiv 1-\exp \left(-{\beta }_{ij}/2\right)$ and the second link’s is the same ${p}_{ji}\equiv 1-\exp \left(-{\beta }_{ji}/2\right)$. It is understood that *Z*_RC_ will only depend on the sum, *β*_*ji*_/2 + *β*_*ij*_/2 = *β*_*ij*_, or the total probability of having at least one link connecting *i* and *j*: $1-\left(1-{p}_{ij}\right)\left(1-{p}_{ji}\right)=1-\exp \left[-\left({\beta }_{ij}+{\beta }_{ji}\right)/2\right]$.

We also introduce *β*_*ii*_ (a self-loop) for every node *i*, which solely serves as a placeholder. This is since whether the self-loop is present or absent does not affect the number of connected components, and thus the summation over configurations of self-loops only contributes to a constant. The complete definition of *β*_*ij*_ for $1\le i,j\le \left|V\right|$ leads to an expansion of our sample space from $\omega \in \Omega={\{0,1\}}^{\left|E\right|}$ to $\omega \in {{\mathcal{B}}}={\{0,1\}}^{\left|V\right|\times \left|V\right|}$, where each realization *ω* now consists of ${\left|V\right|}^{2}$ binary variables, each denoted as *ω*_*ij*_. Taken together, this transformation allows us to rewrite *Z*_RC_ as follows:

31 $${Z}_{{{\rm{RC}}}}=	 {\sum }_{\omega \in {{\mathcal{B}}}}\left[{\prod }_{i,j}^{\left|V\right|}{\left(1-{e}^{-{\beta }_{ij}/2}\right)}^{{\omega }_{ij}}{\left({e}^{-{\beta }_{ij}/2}\right)}^{1-{\omega }_{ij}}\right]{q}^{c(\omega )} \\=	 {\sum }_{\omega \in {{\mathcal{B}}}}\left[{\prod }_{i,j}^{\left|V\right|}{\left({e}^{{\,\beta }_{ij}/2}-1\right)}^{{\omega }_{ij}}\left({e}^{-{\beta }_{ij}/2}\right)\right]{q}^{c(\omega )} \\=	 {\sum }_{\omega \in {{\mathcal{B}}}}\left[{\prod }_{i,j}^{\left|V\right|}{\left({e}^{-{L}_{ij}/2}-1\right)}^{{\omega }_{ij}}\right]{q}^{c(\omega )}.$$

In the last step, we have utilized the fact that

32 $${e}^{-{\sum }_{j}{\beta }_{ij}/2}\equiv {\left({e}^{-{\sum }_{j}\,{\beta }_{ij}/2}-1\right)}^{0}+{\left({e}^{-{\sum }_{j}\,{\beta }_{ij}/2}-1\right)}^{1}$$

to absorb this constant back into the sum over the placeholder self-loop configuration *ω*_*ii*_ ∈ {0, 1}, which leads to the Laplacian diagonal *L*_*ii*_.

It is important to note that in Eq. (31), the realization *ω* represents a directed, loopy graph, and *c*(*ω*) corresponds to the number of weakly connected components of every realization *ω*. Also, the placeholder *β*_*ii*_ no longer appears in the Laplacian after the derivation in Eq. (31). Taken together, this leads us to the formula Eq. (20).

Further, we seek to express ${\mu }_{ij}^{(q)}$ as a derivative of *Z*_RC_ with respect to *L*_*ij*_. Since only $\left|V\right|\left(\left|V\right|-1\right)/2$ variables *β*_*ij*_ are independently defined in the graph Laplacian, it means that varying one such (undirected) link weight *β*_*ij*_ affects four terms of the Laplacian, *L*_*ij*_, *L*_*ji*_, *L*_*ii*_, and *L*_*jj*_, which all depend on *β*_*ij*_. We thus derive the following generalization:

33 $$\frac{\partial }{\partial {\beta }_{ij}}=\frac{\partial }{\partial {L}_{ii}}+\frac{\partial }{\partial {L}_{jj}}-\frac{\partial }{\partial {L}_{ij}}-\frac{\partial }{\partial {L}_{ji}},$$

which, combined with Eq. (29), leads to the formula Eq. (21).

#### Generalization to directed networks

Our formulation in Eqs. (20) and (21) can be immediately generalized to directed networks. This is achieved by replacing the symmetric graph Laplacian with either the outdegree Laplacian [in the same form of Eq. (19)] or the indegree Laplacian,

34 $${L}_{ij}={\sum }_{k}{\beta }_{ki}{\delta }_{ij}-{\beta }_{ij}.$$

When using these directed Laplacians, the resulting connectivity measure ${\mu }_{ij}^{(q)}$ remains symmetric, i.e., ${\mu }_{ij}^{(q)}={\mu }_{ji}^{(q)}$, despite the underlying asymmetry of the network. Indeed, within our formulation, a directed graph having distinct weights *β*_*ij*_ and *β*_*ji*_ is fully equivalent to an undirected graph having an undirected link *e* = {*i*, *j*} with a combined weight, *β*_*e*_ = *β*_*ij*_ + *β*_*ji*_. This fundamental equivalence means one can always convert a directed weighted graph into an equivalent undirected graph by summing the weights *β*_*ij*_ and *β*_*ji*_. For simulation purposes, it is often simpler to perform this conversion first and then apply algorithms designed for undirected weighted graphs.

### RC connectivity in the limit *q* → 0

In the following, we provide proofs for Theorems 1–3 in the main paper.

#### Effective resistance connectivity

##### Proof for Theorem 1

As *q* → 0, the Laplacian scales linearly with the probability associated with each link, ${L}_{ij}={\beta }_{e} \sim {p}_{e} \sim \sqrt{q}/{r}_{e}$. In the *q* → 0 limit, only connected subgraphs contribute to the partition function [Eq. (20)]. In addition, when *L*_*ij*_ → 0 (but slower than the rate of *q* → 0), Eq. (20) can be linearized, and one can show that only the spanning trees, which are connected subgraphs that have the minimum possible number of links ($\left|V\right|-1$), contribute to the partition function *Z*_RC_. Generalizing Kirchhoff’s matrix tree theorem to weighted graphs, it is straightforward to show that ${Z}_{{{\rm{RC}}}}\propto \det ({L}_{+})$, where $\det ({L}_{+})$ is a shorthand for the pseudo-determinant of *L*. Note that $\partial {{\rm{ln}}}\det ({L}_{+})/\partial {L}_{ij}={\left({L}_{+}^{-1}\right)}_{ji}$, where ${L}_{+}^{-1}$ is similarly a shorthand for the pseudo-inverse of *L*. Using the expression of ${\mu }_{ij}^{(q)}$ in terms of the graph Laplacian [Eq. (21)], it leads to

35 $${\mu }_{ij}^{(q)}\simeq 1-q\left[{\left({L}_{+}^{-1}\right)}_{ii}+{\left({L}_{+}^{-1}\right)}_{jj}-{\left({L}_{+}^{-1}\right)}_{ij}-{\left({L}_{+}^{-1}\right)}_{ji}\right],$$

and hence

36 $$\begin{array}{rcl}1/{\nu }_{ij}^{(q)} &=& 1+{q}^{-1}\left(1/{\mu }_{ij}^{(q)}-1\right)\hfill\\ & \simeq & 1+\left[{\left({L}_{+}^{-1}\right)}_{ii}+{\left({L}_{+}^{-1}\right)}_{jj}-{\left({L}_{+}^{-1}\right)}_{ij}-{\left({L}_{+}^{-1}\right)}_{ji}\right]\\ & \simeq & \left[{\left({L}_{+}^{-1}\right)}_{ii}+{\left({L}_{+}^{-1}\right)}_{jj}-{\left({L}_{+}^{-1}\right)}_{ij}-{\left({L}_{+}^{-1}\right)}_{ji}\right]\hfill\\ &=& {R}_{ij}/\sqrt{q},\hfill\end{array}$$

where the third step uses ${L}_{+}^{-1} \sim 1/\sqrt{q}$, and the last step uses the expression of effective resistance in terms of graph Laplacian^19^. Together, this gives rise to Eq. (8) in the main paper. □

#### Shortest path connectivity

##### Proof for Theorem 2

We first consider an unweighted graph (where *l*_*e*_ ≡ 1, so *p*_*e*_ ≡ *p* = *q*^2^) and examine the behavior of ${\mu }_{ij}^{(q)}$ as *q* → 0. Since only configurations *ω* where *i* and *j* are connected contribute to ${\mu }_{ij}^{(q)}$, the first term contributing to the sum [Eq. (4) in the main paper] will be

37 $$\begin{array}{rcl} & \propto & \frac{{p}^{{d}_{ij}}{\left(1-p\right)}^{\left|E\right|-{d}_{ij}}{q}^{\left|V\right|-{d}_{ij}}+\ldots }{{\left(1-p\right)}^{\left|E\right|}{q}^{\left|V\right|}+\ldots }\hfill\\ & \simeq & {\left({q}^{2}\right)}^{{d}_{ij}}{q}^{-{d}_{ij}}+\ldots \hfill\\ &=& {q}^{{d}_{ij}}+\ldots,\hfill\end{array}$$

which corresponds to the configuration(s) *ω* that contains only the shortest path(s) of length *d*_*ij*_ connecting *i* and *j*. Since multiple shortest paths may exist, there could be a prefactor in front of Eq. (37), but this won’t affect the limit.

Now, consider adding one more link to such a shortest path configuration *ω*. This introduces an additional factor of *p* ≃ *q*^2^ from the link addition probability. This new link may also reduce the number of clusters by one, introducing a relative factor *q*^−1^, but the net contribution  ~*q*^2−1^ = *q* will still make the configurations with additional links higher order in *q*. Therefore, the term in Eq. (37) also represents the leading term of ${\mu }_{ij}^{(q)}$ as *q* → 0, and thus Eq. (9) in the main paper holds.

The same argument extends to weighted graphs, provided all link lengths are positively defined (*l*_*e*_ > 0). In this case, the first term in ${\mu }_{ij}^{(q)}$ arises from shortest paths where *d*_*ij*_ = ∑_*e*∈shortest path _*l*_*e*_. Adding an extra link *f* (with length *l*_*f*_) to a shortest path configuration introduces a factor ${p}_{f}={q}^{{l}_{f}+1}$. Combined with a potential *q*^−1^ factor from a cluster count reduction, the net scaling ${q}^{{l}_{f}}$ still ensures that such terms are of higher order in *q*, and thus the shortest path(s) dominates. □

#### Minimum cut connectivity

##### Proof for Theorem 3

We again consider an unweighted graph (where *c*_*e*_ = 1, so *p*_*e*_ ≡ *p* = 1 − *q*) and examine the behavior of $1-{\mu }_{ij}^{(q)}$ as *q* → 0. Since only configurations *ω* where *i* and *j* are disconnected contribute to $1-{\mu }_{ij}^{(q)}$, the first term contributing to $1-{\mu }_{ij}^{(q)}$ will be

38 $$	 \propto \frac{{p}^{\left|E\right|-{\uplambda }_{ij}}{\left(1-p\right)}^{{\uplambda }_{ij}}{q}^{2}+\ldots }{{p}^{\left|E\right|}q+\ldots } \\ 	 \simeq {q}^{{\uplambda }_{ij}}q+\ldots \\ 	={q}^{{\uplambda }_{ij}+1}+\ldots,$$

which corresponds to the configuration(s) *ω* where *i* and *j* have been disconnected by removing a minimum set of *λ*_*ij*_ links (a minimum *i*–*j* cut) from the full graph. Again, since multiple minimum cuts may exist, there could be a prefactor in front of Eq. (38), but this won’t affect the limit.

Now, consider deleting one more link from such a minimum cut configuration *ω*. This introduces an additional factor of 1 − *p* ≃ *q* from the link deletion probability. This deletion will only increase the number of clusters, and the net contribution always makes the configurations with additional deletions higher order in *q*. Therefore, the term in Eq. (38) also represents the leading term of $1-{\mu }_{ij}^{(q)}$ at *q* → 0. Finally, using $\lim\limits_{q\to 0}\left(1-{\mu }_{ij}^{(q)}\right)\simeq q\left(1-{\nu }_{ij}^{(q)}\right)$, we find that Eq. (10) in the main paper holds.

The same argument extends to weighted graphs, provided all link capacities are positively defined (*c*_*e*_ > 0). In this case, the first term in ${\mu }_{ij}^{(q)}$ arises from minimum cuts where *λ*_*ij*_ = ∑_*e*∈minimum cut _*c*_*e*_. Deleting an extra link *f* (with capacity *c*_*f*_ ) from a minimum-cut configuration introduces a factor $1-{p}_{f}={q}^{{c}_{f}}$, which is of higher order in *q* since the number of clusters will only increase. Thus, the minimum cut(s) dominates. □

#### Reinterpretation of classical connectivity measures

The emergence of all aforementioned traditional connectivity metrics in the *q* → 0 limit is not coincidental but highlights a deeper, physical relationship. Indeed, under *q* → 0 (before taking the limit in *p*), configurations that are globally connected, denoted by the set $\omega \in {{\mathcal{C}}}$, dominate the RC ensemble. It is this focus on globally connected graphs ${{\mathcal{C}}}$ that governs classical connectivity metrics.

For example, consider the effective resistance limit *p* ~ *O*(*q*^1/2^) → 0. This condition implies that the subset of ${{\mathcal{C}}}$ that have the minimum number of links become even more dominant. These are precisely the spanning trees of the graph, the count of which is directly related to effective resistance^4^.

On the other hand, while configurations dominant for shortest path and minimum cut measures are no longer globally connected, these can still be understood as derived from the configurations in ${{\mathcal{C}}}$. For shortest paths, the condition *p* ~ *q*^1+*O*(1)^suppresses links and ensures that only the most efficient subgraphs of $\omega \in {{\mathcal{C}}}$, namely shortest paths, remain as dominant contributions. For minimum cuts, where *p* ~ 1 − *q*^*O*(1)^, the dominant configurations represent perturbations to $\omega \in {{\mathcal{C}}}$ that are the most efficient ways to cut *ω* into disconnected graphs.

Taken together, we have shown how the notion of connectivity is reinterpreted in terms of the deeper concept of connectedness, from which the three traditional measures arise as specific, limiting cases. These limits effectively select for configurations that are already globally connected or critically structured as a limiting subset of all possible binary configurations ($\sim {2}^{{\left|V\right|}^{2}/2}$). It remains an open question whether other distinct limits in the *q* → 0 regime^4^ map to other established connectivity measures.

### Series and parallel rules for the RC model

In the main paper, we introduce the series and parallel reduction rules for the RC model. These rules represent a generalization of the rules for standard bond percolation (*q* = 1). These percolation rules are given as follows:

- **Series rule (for percolation)**. If two links *e* and *f* share exactly one common endpoint of degree two (i.e., that node is incident only to *e* and *f*), then they may be contracted into a single link with effective probability
  $$p^{\prime} \,=\,{p}_{e}\,{p}_{f},$$
  and the intermediate node is removed.
- **Parallel rule (for percolation)**. If *e* and *f* connect the same pair of nodes, they may be merged into a single link whose probability is
  $$p^{\prime} \,=\,1-(1-{p}_{e})\,(1-{p}_{f}).$$

Whenever such series or parallel configurations occur, we replace *e* and *f* by a single equivalent link of probability $p^{\prime}$, thereby simplifying the graph.

For general *q*, these rules are generalized by replacing *p*_*e*_ by the dual probabilistic variables, *u*_*e*_ and *v*_*e*_ [Eq. (11) in the main paper]. In what follows, we will illustrate and verify the generalized rules through simple series and parallel configurations.

#### Parallel configuration

Let us check the parallel rule first. Suppose we have two links of presence probabilities *p*_*e*_ and *p*_*f*_ in parallel between two nodes *i* and *j*. The *μ*-connectivity ${\mu }_{ij}^{(q)}$ is given by

39 $${\mu }_{ij}^{(q)}=\frac{{p}_{e}{p}_{f}q+{p}_{e}\left(1-{p}_{f}\right)q+\left(1-{p}_{e}\right){p}_{f}q}{{p}_{e}{p}_{f}q+{p}_{e}\left(1-{p}_{f}\right)q+\left(1-{p}_{e}\right){p}_{f}q+\left(1-{p}_{e}\right)\left(1-{p}_{f}\right){q}^{2}},$$

in which the denominator includes all four possible configurations where the two independent links are present or absent, each multiplied by a factor *q* ^*c*(*ω*)^ where *c*(*ω*) is the number of clusters given the configuration *ω*. The numerator in Eq. (39) includes the three cases where nodes *i* and *j* are connected. We then define *v*_*e*_ = *p*_*e*_ and *v*_*f*_ = *p*_*f*_ as in the main paper. After some rearrangement, we find

40 $${\mu }_{ij}^{(q)}=\frac{{\nu }_{ij}^{(q)}q}{{\nu }_{ij}^{(q)}q+\left(1-{\nu }_{ij}^{(q)}\right){q}^{2}},$$

where

41 $${\nu }_{ij}^{(q)}=1-\left(1-{v}_{e}\right)\left(1-{v}_{f}\right)$$

is precisely the parallel rule for the *ν*-connectivity. As explained in the main paper, ${\nu }_{ij}^{(q)}$ is the effective probability before taking the final *q*^*c*(*ω*)^ factor into account: with probability ${\nu }_{ij}^{(q)}$, nodes *i* and *j* are connected, for which *c*(*ω*) = 1; with probability $1-{\nu }_{ij}^{(q)}$, nodes *i* and *j* are disconnected, for which *c*(*ω*) = 2. This explains the form of Eq. (40).

#### Series configuration

Next, we check the series rule. Suppose we have two links of presence probabilities *p*_*e*_ and *p*_*f*_ in series, connecting three nodes (*i* to *k* to *j*). Similarly, the *μ*-connectivity ${\mu }_{ij}^{(q)}$ is given by

42 $${\mu }_{ij}^{(q)}=\frac{{p}_{e}{p}_{f}q}{{p}_{e}{p}_{f}q+{p}_{e}\left(1-{p}_{f}\right){q}^{2}+\left(1-{p}_{e}\right){p}_{f}{q}^{2}+\left(1-{p}_{e}\right)\left(1-{p}_{f}\right){q}^{3}},$$

After some rearrangement, we find

43 $$\begin{array}{r}{\mu }_{ij}^{(q)}={u}_{e}^{(q)}{u}_{f}^{(q)},\end{array}$$

where *u*_*e*_ satisfies

44 $$\begin{array}{r}{u}_{e}=\frac{{p}_{e}q}{{p}_{e}q+\left(1-{p}_{e}\right)\,{q}^{2}},\end{array}$$

and the same for *u*_*f*_ :

45 $$\begin{array}{r}{u}_{f}=\frac{{p}_{f}q}{{p}_{f}q+\left(1-{p}_{f}\right)\,{q}^{2}}.\end{array}$$

This is precisely the series rule for the *μ*-connectivity.

#### Proof of the path redundancy inequality

We begin by expressing the inequality (13) in the main paper directly in terms of *u*_*e*_, *u*_*f*_, and *u*_*g*_. The left-hand side of the inequality can be rewritten as

46 $$	 {{\rm{para}}}\{\,1-{{\mathcal{M}}}({{\rm{seri}}}\{{u}_{e},{u}_{f}\})\,,\,{v}_{g}\} \\ 	=1-(1-{v}_{g})\,{{\mathcal{M}}}({u}_{e}\,{u}_{f}) \\ 	=1-{{\mathcal{M}}}({u}_{g})\,{{\mathcal{M}}}({u}_{e}\,{u}_{f});$$

while the right-hand side is

47 $$	 1-{{\mathcal{M}}}\left({{\rm{seri}}}\{{u}_{e},{{\mathcal{M}}}(1-{{\rm{para}}}\{{v}_{f},{v}_{g}\})\}\right) \\ 	=1-{{\mathcal{M}}}\left(\,{u}_{e}\,{{\mathcal{M}}}\left((1-{v}_{f})(1-{v}_{g})\right)\right) \\ 	=1-{{\mathcal{M}}}\left(\,{u}_{e}\,{{\mathcal{M}}}\left({{\mathcal{M}}}({u}_{f})\,{{\mathcal{M}}}({u}_{g})\right)\right).$$

The inequality thus becomes

48 $$1-{{\mathcal{M}}}({u}_{e}\,{u}_{f})\,{{\mathcal{M}}}({u}_{g}) > 1-{{\mathcal{M}}}\left({u}_{e}\,{{\mathcal{M}}}({{\mathcal{M}}}({u}_{f})\,{{\mathcal{M}}}({u}_{g}))\right).$$

After simplification, the inequality (48) becomes

49 $$\frac{ED}{ABC} < 0,$$

where

$$	 A=1+(q-1){u}_{e}{u}_{f},\\ 	 B=1+(q-1){u}_{g},\\ 	 C=1+(q-1)\left[{u}_{f}{u}_{g}+{u}_{e}\left({u}_{f}+{u}_{g}+(q-2){u}_{f}{u}_{g}\right)\right],\\ 	 D=1+(q-1){u}_{f}\left[{u}_{g}+{u}_{e}\left({u}_{f}+{u}_{g}+(q-2){u}_{f}{u}_{g}\right)\right],\\ 	 E=q({u}_{e}-1){u}_{g}.$$

Since 0 < *u*_*e*_, *u*_*f*_, *u*_*g*_ < 1 and *q* > 0, the factor *E* is strictly negative, while *A* and *B* are strictly positive. Hence, it suffices to show

50 $$\frac{D}{C} > 0.$$

To this end, we compute the derivatives:

51 $$\frac{d C}{d q}={u}_{f}{u}_{g}+{u}_{e}{u}_{f}+{u}_{e}{u}_{g}-3{u}_{e}{u}_{g}{u}_{f}+2q{u}_{e}{u}_{f}{u}_{g},$$

52 $$\frac{d D}{d q}={u}_{f}({u}_{g}+{u}_{e}{u}_{f}+{u}_{e}{u}_{g}-3{u}_{e}{u}_{g}{u}_{f}+2q{u}_{e}{u}_{f}{u}_{g}).$$

Setting each derivative to zero gives the critical values

53 $${q}_{C}^{ * }=\frac{3}{2}-\frac{1}{2{u}_{e}}-\frac{1}{2{u}_{f}}-\frac{1}{2{u}_{g}},$$

54 $${q}_{D}^{ * }=\frac{3}{2}-\frac{1}{2{u}_{f}{u}_{e}}-\frac{1}{2{u}_{f}}-\frac{1}{2{u}_{g}}.$$

Since 0 < *u*_*e*_, *u*_*f*_, *u*_*g*_ < 1, we have ${q}_{C}^{ * } < 0$ and ${q}_{D}^{ * } < 0$. It follows that given *q* > 0, both *C* and *D* are strictly increasing in *q*. Then, evaluating at *q* → 0 yields

55 $$C > C(0)=1-\left[{u}_{f}{u}_{g}+{u}_{e}({u}_{f}+{u}_{g}-2{u}_{f}{u}_{g})\right],$$

56 $$D > D(0)=1-{u}_{f}\left[{u}_{g}+{u}_{e}({u}_{f}+{u}_{g}-2{u}_{f}{u}_{g})\right].$$

Note *u*_*f*_ + *u*_*g*_ − 2*u*_*f*_*u*_*g*_ > 0. Thus, the quantities *u*_*f*_*u*_*g*_ + *u*_*e*_(*u*_*f*_ + *u*_*g*_ − 2*u*_*f*_*u*_*g*_) and *u*_*f*_[*u*_*g*_ + *u*_*e*_(*u*_*f*_ + *u*_*g*_ − 2*u*_*f*_*u*_*g*_)] are both strictly increasing in *u*_*e*_ on *u*_*e*_ ∈ (0, 1). Consequently, the lower bounds

57 $$1-\left[{u}_{f}{u}_{g}+{u}_{e}({u}_{f}+{u}_{g}-2{u}_{f}{u}_{g})\right],{{\rm{and}}}$$

58 $$1-{u}_{f}\left[{u}_{g}+{u}_{e}({u}_{f}+{u}_{g}-2{u}_{f}{u}_{g})\right],$$

are strictly decreasing in *u*_*e*_. Now, setting *u*_*e*_ = 1 in the first bound gives

59 $$C > 1-({u}_{f}{u}_{g}+{u}_{f}+{u}_{g}-2{u}_{f}{u}_{g})\\=(1-{u}_{f})(1-{u}_{g}) > 0.$$

Similarly, setting *u*_*e*_ = 1 in the second bound yields

60 $$D > 1-{u}_{f}({u}_{g}+{u}_{f}+{u}_{g}-2{u}_{f}{u}_{g})\\=(1-{u}_{f})(1+{u}_{f}-2{u}_{f}{u}_{g}) > (1-{u}_{f})^2 > 0.$$

We thus find *C* > 0 and *D* > 0, and hence the left-hand side of the inequality (49) is negative. □

### Random-walk interpretation of effective resistance

Given a homogeneous network with ${p}_{e}\equiv p=\sqrt{q}$, the partition function *Z*_RC_ will be dominated in the *q* → 0 limit by uniform spanning trees (USTs), where each distinct spanning tree of the network is assigned an equal probability of presence. The USTs are deeply connected to the effective resistance. Consider a link {*i*, *j*}, the effective resistance *R*_*ij*_ is known as the probability that the link {*i*, *j*} is chosen in a randomly sampled UST^45^. This can be seen using the definition of conditional probability: Let the events *A* = {link {*i*, *j*} is present} and *B* = {*i* is connected to *j*}. We have $P(A)=p=\sqrt{q}$ and $P(B)={\nu }_{ij}^{(q)}$. Since *A* ⊂ *B*, the conditional probability

61 $$\begin{array}{r}P(A| B)=P(A)/P(B)=\sqrt{q}/{\nu }_{ij}^{(q)}\end{array}$$

becomes the probability of having the link {*i*, *j*} in a UST in the *q* → 0 limit. By Eq. (8) in the main paper, this limit is indeed equal to the effective resistance *R*_*ij*_.

Given the correspondence between effective resistance and random walk^25^, there is also a well-established relationship that connects UST to random walk: the formation of a UST is deeply linked to paths traced by a single random walker. To be specific, following the Aldous–Broder algorithm^51,52^, a link {*i*, *j*} is present in a randomly chosen UST if and only if when a random walk first reaches node *j*, it does so by traversing the link {*i*, *j*} from *i* to *j*. This random-walk relationship provides additional insight into the connection between ${\nu }_{ij}^{(q)}$ and effective resistance *R*_*ij*_. Consider, again, a given link {*i*, *j*} in the network. We have argued that in the $p=\sqrt{q}\to 0$ limit, $\sqrt{q}/{\nu }_{ij}^{(q)}={R}_{ij}$ is equal to the probability of having link {*i*, *j*} in the UST. Therefore, based on the Aldous–Broder algorithm, this probability is also equal to the probability that a random walk started at *i* reaches *j* for the first time, specifically via {*i*, *j*}. This provides a random-walk interpretation for the effective resistance measure.

### Correlations in network dynamics

#### Epidemic model (SIR)

We considered a stochastic Susceptible-Infected-Recovered (SIR) compartmental model on a network^50^. In this model, initially, all nodes are in the susceptible (S) state, with the exception of a single seed node, *j*, which is set to the infected (I) state. The infection then proceeds in discrete time steps. At each step, every infected node attempts to transmit the disease to each of its susceptible (uninfected) neighbors. The transmission along any single link from an infected node to a susceptible neighbor occurs with probability *p*. If a susceptible node *i* is connected to multiple infected neighbors, it can be infected through any of these links. The probability that node *i* becomes newly infected at a given time step, assuming it was susceptible, is therefore given by

62 $${{\rm{Prob}}}({{\rm{node}}}\,i\,{{\rm{becomes}}}\,{{\rm{infected}}})=1-{\left(1-p\right)}^{{k}_{i}(I)},$$

where *k*_*i*_(*I*) is the number of currently infected neighbors of node *i*. Furthermore, letting $p\equiv 1-\exp \left(-\beta \right)$, this probability can be expressed in terms of either the adjacency matrix (the typical formulation^57^) or, to align with our framework, the weighted graph Laplacian:

63 $${{\rm{Prob}}}({{\rm{node}}}\,i\,{{\rm{becomes}}}\,{{\rm{infected}}})=1-\exp \left(-{\sum }_{i\ne l}{L}_{il}{I}_{l}\right),$$

where *L*_*il*_ ≡ *β* when *i* and *l* are two end nodes of a link that has a uniform link weight *β*. Here, *I*_*l*_ = 1 if node *l* is currently infected, and *I*_*l*_ = 0 otherwise. Then, in the next time step, all nodes that were infected transition to the recovered (R) state and can no longer participate in the infection process (i.e., they cannot infect others or be reinfected).

This stochastic simulation is run for a sufficiently long duration and repeated many times for each chosen initial seed node *j*. Averaging all simulations, we can determine Ξ_*ij*_(*t*), the probability that node *i* is in the recovered state at time *t*, given that node *j* was the initial seed node (note that the order in the tuple (*i*, *j*) matters). The quantity Ξ_*ij*_(*t*) serves as a measure of the correlation between nodes *i* and *j* under this SIR process, reflecting how readily an infection starting at *j* can reach and affect *i* after duration *t*. Our central hypothesis is that this dynamically generated correlation, Ξ_*ij*_(*t*), can be effectively predicted by the RC connectivity measure ${\nu }_{ij}^{(q)}$, derived purely from the network structure and the RC parameters.

#### Neurodynamic model (FHN)

We also employed the FitzHugh–Nagumo (FHN) model^61,62^ (a simplification of the Hodgkin–Huxley model^63^) for describing neuronal action potentials subject to physical constraints^64^. The FHN equations describe the evolution of neuron membrane voltage *v* and an auxiliary, slower recovery variable *w*:

64 $$	 \dot{v}=v-\frac{{v}^{3}}{3}-w+\left({\nabla }^{2}v+{I}_{{{\rm{ext}}}}\right),\\ 	 \tau \dot{w}=v+a-bw.$$

Here, the second-order spatial derivative ∇^2^*v* represents the spatial diffusion of voltage, reflecting current propagation along an axon as described by the cable theory^65^. When considering a network of *N* neurons, each node *i* possesses its own voltage *v*_*i*_(*t*) and recovery variable *w*_*i*_(*t*). The spatial coupling term ∇^2^*v*_*i*_ is then replaced by a sum over contributions from connected neurons,  − ∑_*l*_*L*_*il*_*v*_*l*_, modeled using the weighted graph Laplacian. The Laplacian’s uniform magnitude *L*_*il*_ ≡ *β* (when *i* and *l* share a link) sets the strength of internodal coupling, effectively reflecting the diffusion constant in the system. The parameters governing the recovery variable *w*_*i*_ are set to standard, node-independent values: *a* = 0.7, *b* = 0.8, and *τ* = 12.5.

To probe the correlations, the system is initialized with all external currents set to zero except for a single chosen node *j*. For this node *j*, a constant external current *I*_ext,*j*_(*t*) ≡ *I* is applied. Under appropriate conditions (e.g., *I* in an excitable regime), this sustained stimulus can drive the network into a state where nodes exhibit oscillatory voltage dynamics.

After the system settles into a statistically stationary oscillatory state, we measure the dynamics of each node *i* by defining ${{{\mathcal{V}}}}_{ij}(I)$ as the peak-to-peak value of the voltage oscillations of node *i*, given that node *j* was the site of the sustained external current *I*. A larger value of ${{{\mathcal{V}}}}_{ij}(I)$ indicates a stronger influence of the activity initiated at *j* on the dynamics of node *i*. Again, we propose that this dynamically generated correlation, ${{{\mathcal{V}}}}_{ij}(I)$, can be effectively predicted by the network structure using the RC connectivity ${\nu }_{ij}^{(q)}$.

#### Simulation

We investigated network dynamics on Zachary’s karate club, a known benchmark network comprising 34 nodes and 78 links, which famously exhibits a split into two distinct communities^66^. As demonstrated previously, the governing equations for both the SIR and FHN models can be formulated in terms of the graph Laplacian, whose construction incorporates the link weights *β*. This formulation allowed us to simulate these two dynamical models by systematically varying *β*.

For the SIR model, we computed the correlation function Ξ_*ij*_(*t*) for every tuple (*i*, *j*) by averaging results from 1 × 10^6^ stochastic simulations. The per-link infection probability *p* (used in the SIR dynamics) was directly determined from the link weight *β* via the Edwards–Sokal relation 1 − *p* ≡ *e*^−*β*^. For the FHN model, we computed the relevant measure ${{{\mathcal{V}}}}_{ij}(I)$ for every tuple (*i*, *j*) over a simulation time from *t* = 0 to *t* = 200, using a discrete time step of Δ*t* = 0.001. The peak-to-peak amplitude was then determined within the interval *t* ∈ [100, 200], by which time the system’s oscillations had stabilized. The external current *I* and the link weight *β* (representing the diffusion constant in this context) were chosen to ensure that the system operated in the excitable regime.

We aim to demonstrate that both correlation functions Ξ_*ij*_(*t*) (from SIR) and ${{{\mathcal{V}}}}_{ij}(I)$ (from FHN) can be effectively predicted by the RC connectivity measure (note that both Ξ_*ij*_(*t*) and ${{{\mathcal{V}}}}_{ij}(I)$ are inherently asymmetric matrices). To this end, we calculated the RC connectivity, ${\nu }_{ij}^{(q)}$ analytically from the graph Laplacian *L*_*ij*_.

**Fig. 6:**
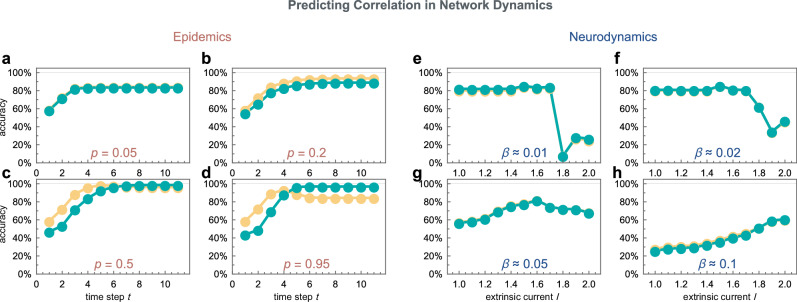
Network dynamics captured by RC connectivity (*q* = 0.0025). **a**–**h** Same settings as Fig. 4 but with *q* = 0.0025.

**Fig. 7:**
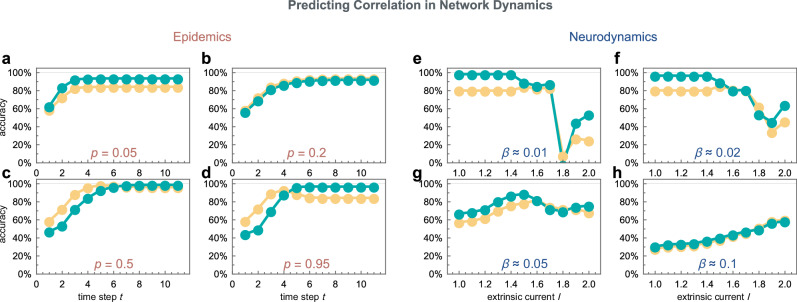
Network dynamics captured by RC connectivity (*q* = 0.1). **a**–**h** Same settings as Fig. 4 but with *q* = 0.1.

**Fig. 8:**
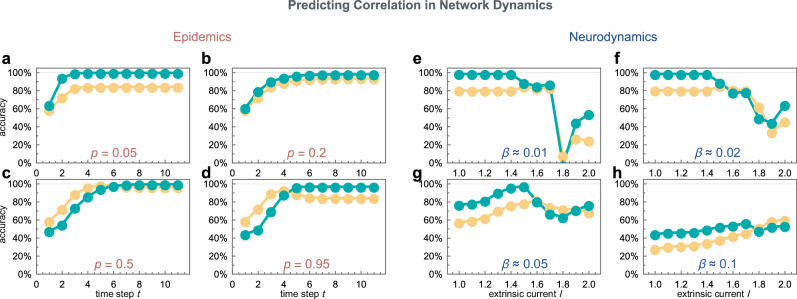
Network dynamics captured by RC connectivity (*q* = 0.5). **a**–**h** Same settings as Fig. 4 but with *q* = 0.5.

**Fig. 9:**
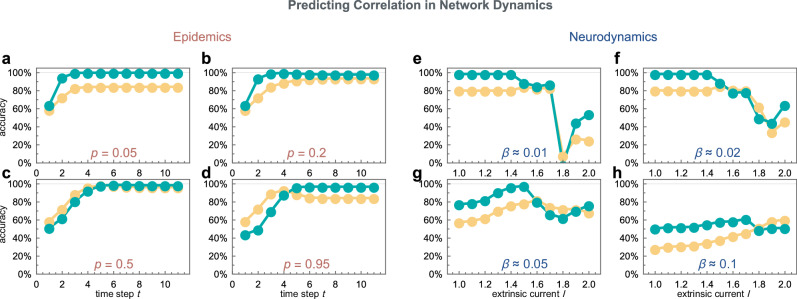
Network dynamics captured by RC connectivity (*q* = 2). **a**–**h** Same settings as Fig. 4 but with *q* = 2.

In addition to Fig. 4, we also examined the dependence of ${\mu }_{ij}^{(q)}$ on *q*. As Figs. 6–9 show, a small value (*q* = 0.0025) typically yields worse results compared to a larger value (*q* = 2). This suggests that tuning *q* is essential for capturing the path interactions in these network dynamical models. Notably, in the limit ${\beta} \sim p \sim \sqrt{q}\to 0$, the *μ*-connectivity reduces to the effective resistance (e.g., Fig. 6a).

### Dominance by degree and modularity

**Fig. 10:**
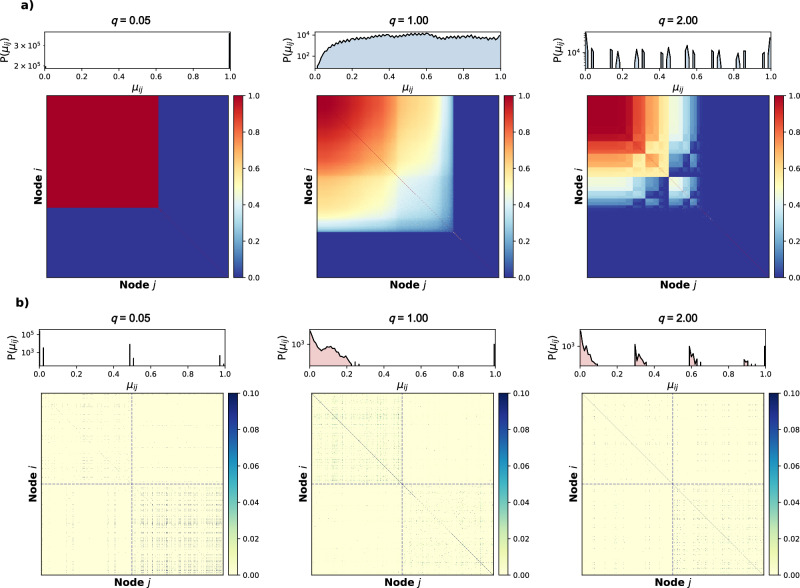
Distribution of RC connectivity. Distributions *P*(*μ*_*ij*_) and connectivity matrices *μ*_*ij*_ are shown for different *q-*values. **a** Link weight *p*_*e*_ ≈ 0.632. Nodes are sorted by descending degree in the matrices. **b** Link weight *p*_*e*_ = 0.10. Nodes are grouped by community in the matrices.

Here, we provide a granular analysis of how the competition between community modularity and node degree centrality shapes observed connectivity patterns. To this end, we examined both the distribution *P*(*μ*_*ij*_) of the RC connectivity values (top of each panel) and the connectivity matrix *μ*_*ij*_ itself (bottom of each panel) in Fig. 10.

We first consider the unweighted case (*p*_*e*_ = 1 − *e*^−1^ ≈ 0.632). In Fig. 10a, as *q* increases (left to right), *P*(*μ*_*ij*_) shifts from a bimodal distribution—where high-degree nodes share *μ*_*ij*_ ≈ 1 while low-degree nodes remain at *μ*_*ij*_ ≈ 0—to a more nuanced profile where degree correlations also shape *μ*_*ij*_. Note that node indices *i*, *j* are already sorted by degree in descending order. In contrast, for smaller link weights (*p*_*e*_ = 0.10), the *μ*_*ij*_ matrix becomes more sensitive to *q* (Fig. 10b). At low *q*, non-zero *μ*_*ij*_ entries correspond to both intra- and inter-community pairs (the node indices *i*, *j* are now grouped by community). At *q* = 1, inter-community entries almost vanish, while small but non-negligible *μ*_*ij*_ values persist between intra-community nodes regardless of degree (consistent with Fig. 5f). Finally, for *q* = 2, the two communities remain robustly defined, while inter-community connectivity is restricted exclusively to global hubs. This indicates that in this regime, community assignment and node degree act simultaneously as potent drivers of the observed connectivity.

## Supplementary information

Transparent Peer Review file

## Data Availability

The generated data are available at: https://github.com/x-meng-group/RandomClusterConnectivity.

## Code availability

The code used for this manuscript is available at: https://github.com/x-meng-group/RandomClusterConnectivity.
