# Supplementary material for: Unifying network connectivity from geodesics to random walks via the random cluster model
Source: Nat Commun. 2026 Jun 27;17:8187. doi: 10.1038/s41467-026-72587-2 (PMC13462334; doi:10.1038/s41467-026-72587-2)
Supplement: Supplementary file 1 [file 41467_2026_72587_MOESM1_ESM.pdf]

nature portfolio

Peer Review File

Unifying Network Connectivity from Geodesics to Random Walks via the Random Cluster Model

Corresponding Author: Professor Renaud Lambiotte

This manuscript has been previously reviewed at another journal. This document only contains information relating to versions considered at Nature Communications.

This file contains all reviewer reports in order by version, followed by all author rebuttals in order by version.

Version 0:

Reviewer comments:

Reviewer #2

(Remarks to the Author)
As stated in my previous report, the paper has demonstrated that the shortest paths, effective resistance, and minimum cut measures can be interpreted as limiting cases of the random cluster model. The paper is well organised and written. The theoretical results are interesting.

The revised version improved the presentation, and the newly added sections help the reader better understand the paper's contribution. The new sections also provide more detail, supporting the claims made in the paper, which were my main concern in the previous version.

Despite that, it would be useful for the reader if the authors shared their code. Given the nature of this work, sharing the code would greatly improve reproducibility and could broaden the paper's impact. Finally, it would be interesting if the authors also provide an analysis of the computational complexity of their methods. Section 2.3, "Implementing RC Connectivity," already provided a description of the algorithms, but step 3 relies on a Monte Carlo simulation, which makes it difficult to assess the actual computational cost of RC connectivity.

Thus, mainly due to the theoretical results in the paper, I would recommend publication of the paper with the above-mentioned minor corrections.

Reviewer #3

(Remarks to the Author)
The authors appositely addressed my concerns from their Nature Physics submissions. I do not have further comments. After considering the Nature Communication's publication standard, I consider this one deserves publication as is.

Open Access This Peer Review File is licensed under a Creative Commons Attribution 4.0 International License, which permits use, sharing, adaptation, distribution and reproduction in any medium or format, as long as you give appropriate credit to the original author(s) and the source, provide a link to the Creative Commons license, and indicate if changes were made.
In cases where reviewers are anonymous, credit should be given to 'Anonymous Referee' and the source.
The images or other third party material in this Peer Review File are included in the article's Creative Commons license, unless indicated otherwise in a credit line to the material. If material is not included in the article's Creative Commons license and your intended use is not permitted by statutory regulation or exceeds the permitted use, you will need to obtain permission directly from the copyright holder.
To view a copy of this license, visit https://creativecommons.org/licenses/by/4.0/

March 10, 2026

Re:
Title: Unifying Network Connectivity: From Geodesics to Random Walks via the Random Cluster Model
To: Prof. Iryna Omelchenko, Senior Editor, Nature Communications.

Dear Prof. Iryna Omelchenko,

Thank you for your email and for the positive decision on our manuscript "Unifying Network Connectivity: From Geodesics to Random Walks via the Random Cluster Model". We are delighted that our work has been **accepted in principle for publication in Nature Communications**.

Reviewer 2:

"The paper is well organised and written. The theoretical results are interesting....."

"mainly due to the theoretical results in the paper, I would recommend publication of the paper....."

We particularly appreciate that the Reviewer recognizes the work as elegant and conceptually and technically interesting.

Reviewer 3:

"The authors appositely addressed my concerns from their Nature Physics submissions. I do not have further comments. After considering the Nature Communication's publication standard, ***I consider this one deserves publication as is.*** *"*

We are extremely grateful for the time and effort the Reviewers have dedicated to our work, as well as their thoughtful and constructive feedback. In the following, we provide a point-by-point response (highlighted in blue) to the reviewers' comments (in *italics*).

Yours sincerely (in the name of all authors),

Renaud Lambiotte	Xiangyi Meng	Gaogao Dong
Mathematical Institute	Department of Physics, Applied Physics, and Astronomy	School of Mathematical Sciences
University of Oxford	Rensselaer Polytechnic Institute<br>Troy, NY 12180, USA	Jiangsu University

Response to Editors' Comments:

We ask you to revise your paper to address our editorial requests (in the attached Author Checklist)

Failure to comply with our editorial requests will result in further revisions and delays in accepting your manuscript. Please also see the Nature Communications formatting instructions for further information.

We have revised the manuscript to address all editorial requests and have carefully completed the Author Checklist, which is attached for your reference.

Response to Reviewers' Comment:

Reviewer 2

Despite that, it would be useful for the reader if the authors shared their code. Given the nature of this work, sharing the code would greatly improve reproducibility and could broaden the paper's impact. Finally, it would be interesting if the authors also provide an analysis of the computational complexity of their methods. Section 2.3, "Implementing RC Connectivity," already provided a description of the algorithms, but step 3 relies on a Monte Carlo simulation, which makes it difficult to assess the actual computational cost of RC connectivity.

We are grateful for the Reviewer's thoughtful assessment and the time invested in evaluating our work. In the revised manuscript, we have included a link to the code repository for reproducibility: https://github.com/x-meng-group/RandomClusterConnectivity.

Additionally, we have updated the computational complexity analysis for all methods, with the changes highlighted in red in the text for your convenience.
